# Artificial intelligence and anti-cancer drugs: advancing discovery, personalization, and precision oncology

**DOI:** 10.3389/fonc.2026.1885312

**Published:** 2026-10-13

**Authors:** Faizan ul Haque Nagrami, Pahchan Shukla, Ruchi Yadav, Jyoti Prakash

**Affiliations:** 1 College of Biotechnology, U.P. Pt. Deen Dayal Upadhyaya Pashu Chikitsa Vigyan Vishwavidyalaya Evam Go Anusandhan, Sansthan (DUVASU), Mathura, India; 2 Amity Institute of Biotechnology, Amity University Uttar Pradesh, Malhaur, Lucknow, India

**Keywords:** artificial intelligence (AI), cancer, drug discovery, multi-omics, precision oncology, validation

## Abstract

Artificial intelligence (AI) is changing the way we discover anticancer drugs. It tackles the problems of traditional methods, such as prolonged timelines for drug development, higher costs, and substantial inter-patient heterogeneity. This review explains how AI is applied throughout the cancer drug pipeline. This includes target identification, virtual screening of compounds, molecular docking, and predicting pharmacokinetics. We explore how machine learning models speed up hit-to-lead optimization, improve clinical trial design by stratifying patients, and allow for real-time toxicity monitoring. A key focus is on integrating AI with multi-omics data, including proteomics, genomics, transcriptomics, and metabolomics, to support precision oncology and personalized treatments. We address important validation challenges, like model interpretability, data quality, and regulatory issues that must be solved before AI can be widely used in clinics. By summarizing current evidence and targeting research gaps, this review offers a clear view of how AI is enhancing the discovery, personalization, and validation of anticancer treatments.

## 1 Introduction

Cancer is a major global health issue marked by abnormal, uncontrolled cell growth that can spread to other organs. It is extremely complex, with genetic variations and the ability to adapt and evolve. It remains a leading cause of mortality worldwide, responsible for approximately 10 million deaths in 2020 (1). Developing effective anticancer drugs is very challenging because of complex molecular interactions, varying patient responses, and the generation of drug resistance (2). Traditional drug discovery methods took more time, resources, and cost anywhere from millions to billions of dollars (3). As a result, there is a pressing need for computational methods to make drug discovery faster and cheaper. Computer-aided and AI-based technologies have changed the scenario of drug discovery and cancer research as they have improved survival predictions, detection, and treatment effectiveness (4). It allows for precise target identification (5), speeds up virtual compound screening and molecular design (6, 7), predicts pharmacokinetics and toxicity, and aids in precision oncology by integrating multi-omics data for precise treatment (5, 8). AI also makes clinical trials more efficient through patient stratification, adaptive designs, and real-time monitoring of drug safety (5). However, despite these advances, ongoing challenges restrict real-world clinical impact. These challenges include fragmented multi-omics data, insufficient external validation of AI models, inconsistent evaluation practices, and a lack of prospective, multicenter studies of AI-created candidates and predictions (5). Many existing reviews discuss AI techniques in oncology but do not clearly differentiate between methodological potential and proven clinical use (9). They also fail to provide practical pathways for transitioning from discovery to clinical application. This review offers a targeted, critical review of how AI is progressing anticancer drug discovery and precision oncology. The aim is to identify where AI shows clear benefits compared to areas where validation issues hinder clinical use. The focus includes computational target nomination and resistance prediction, virtual screening and data-driven molecular design, predicting ADMET and toxicity, integrating genomics, transcriptomics, proteomics, and metabolomics for patient stratification, and designing AI-enabled clinical trials and monitoring drug safety in real-time. Important themes include model interpretability, dataset standardization, external and prospective validation, and readiness for regulation. Our unique contribution is a framework that connects methodological advances with their validation status, points out specific gaps for multicenter testing, and provides recommendations for developers, clinicians, and regulators to promote responsible adoption. By summarizing current findings and identifying research gaps, this review supports the goal of safe, effective, and personalized cancer care through AI-driven discovery, personalization, and validation of anticancer treatments.

## 2 Artificial intelligence in drug discovery

### 2.1 Artificial intelligence methodologies and roles

Artificial Intelligence methodologies, including machine learning (ML), deep learning (DL), reinforcement learning (RL), and natural language processing (NLP), enable rapid analysis of large chemical and biological datasets, prediction of molecular properties, hypothesis generation, and automated molecule design (6, 10). They facilitate key roles in the pipeline: NLP/ML for data curation and integration; ML/DL for target and biomarker nomination; DL and physics-informed models for virtual screening and docking; VAEs, GANs, RL for *de novo* molecule generation and optimization; ADMET and clinical outcomes for translational prediction (refer **Figure 1**).

**Figure 1:**
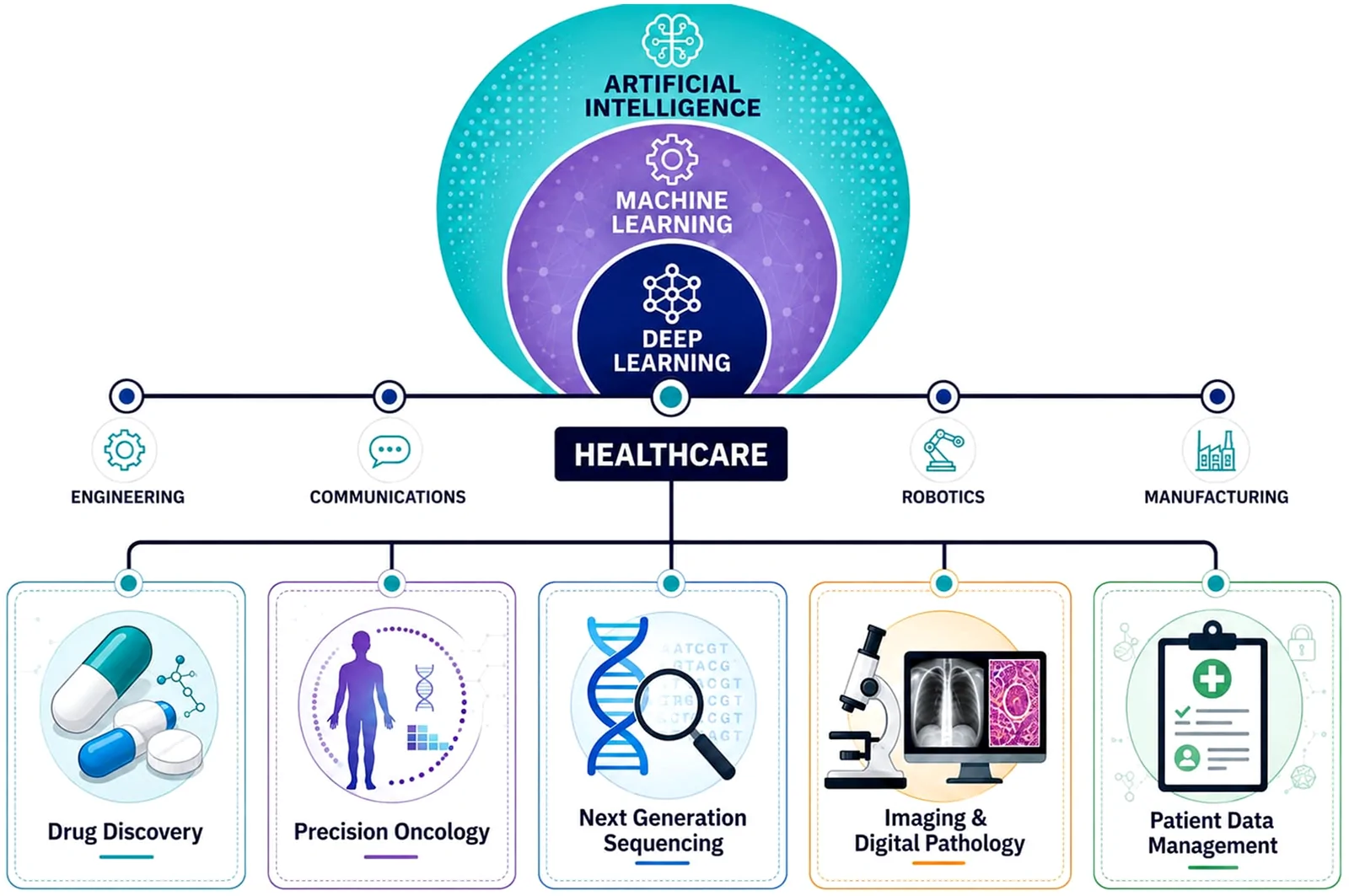
An overview of the applications of artificial intelligence. Source adapted from: Dlamini et al. (11). Recreated by Canva, Luna (https://lunaaiart.app), and BioRender (https://www.biorender.com).

### 2.2 Platforms and tools in drug discovery

A wide range of AI platforms and tools exemplify this transformation. IBM Watson and Benevolent AI assist in data analysis and target identification; DeepMind’s AlphaFold has revolutionized protein structure prediction; Atomwise and Schrödinger employ deep learning and computational chemistry for molecular interaction prediction; and Chemistry42 is a platform for *de novo* small-molecule design and optimization. The InClinico module is used for clinical trial outcome prediction; OpenAI’s GPT models support hypothesis generation and text mining, while NVIDIA Clara provides infrastructure for genomics and medical imaging (10). Structural ML Tools (Schrödinger, Atomwise) do well when high-quality structural or assay data are available; generative design platforms (Chemistry42) rapidly explore novel chemotypes but need downstream filters for synthetic feasibility and ADMET; and NLP/LLM tools excel at literature mining yet require imposition of domain constraints to avoid hallucinations.

### 2.3 Pipeline integration and complementary technologies

These technologies collectively support the pipeline from hit identification and *de novo* molecular design to ADMET prediction, target validation, and drug repurposing. Synergies with complementary innovations, such as CRISPR-based functional genomics, quantum computing for complex simulations, and high-throughput screening, can enhance both speed and precision in discovery workflows (10).

### 2.4 Generative models and reinforcement learning

AI-based models (variational autoencoders, generative adversarial networks, flow models) and reinforcement learning can create novel chemical structures optimized for multiple properties, accelerating discovery beyond the limits of existing libraries. However, many reports focus on internal optimization metrics; robust translational value requires coupling generation with synthetic-accessibility scoring, ADMET filtering, uncertainty estimation, and prospective experimental validation. **Table 1** provides an overview of Representative AI-discovered candidates currently in Clinical Trials and their validation levels.

**Table 1:** An overview of representative AI-discovered drugs in clinical trials.

S.No	Drug	Sponsor	Indication	Phase	NCT identifier
1.	REC-2282 (12)	Recursion	Neurofibromatosis type 2	II/III	NCT05130866
2.	LP-300 (8)	Lantern Pharma	Advanced lung adenocarcinoma	II	NCT05456256
3.	REC-994 (13)	Recursion	Cerebral cavernous malformation	II	NCT05085561
4.	NDI-1150-101 (14)	Nimbus	Solid tumor	I/II	NCT05128487
5.	ERAS-007 (15)	Erasca	Advanced gastrointestinal malignancies	I/II	NCT05039177
6.	REC-4881 (1)	Recursion	Familial adenomatous polyposis	I/II	NCT05552755
7.	LP-184 (16)	Lantern	Solid tumor	I/II	NCT05933265
8.	RLY-4008 (17)	Elevar Therapeutics	Solid tumor	I/II	NCT04526106
9.	SGR-1505 (18)	Schrödinger	Relapsed/refractory B-cell lymphomas	I	NCT05544019
10.	NX-13 (19)	Landos Biopharma	Ulcerative colitis	I	NCT04862741
11.	PHI-101 (20)	Seoul National University Hospital	Acute myeloid leukaemia	I	NCT04842370
12.	RLY-1971 (21)	Hoffmann-La Roche	Metastatic solid tumor	I	NCT04252339
13.	RLY-2608 (22)	Relay Therapeutics	Advanced solid tumor or breast cancer	I	NCT05216432

Source: Adapted and reproduced from Ali et al., (2025) (23) on A New Era of Artificial Intelligence (AI): Transforming Drug Discovery and Nicolaou (24) on Development & Advancing the drug discovery and development process.

### 2.5 ADMET prediction and in silico platforms

ADMET (absorption, distribution, metabolism, excretion, and toxicity) is an integral factor in determining the safety and efficacy of a candidate. In silico ADMET prediction improves attrition rates at an early stage (5). Many ADMET endpoints have been predicted with AI models that use molecular descriptors or fingerprints (RF, SVM, gradient-boosted trees, DL). Although industrial in silico ADMET platforms (like Bayer pipelines with RF and SVM using extended connectivity fingerprints) accelerate triage, external benchmarking and prospective validation on novel chemotypes are still limited. Consensus modeling, uncertainty quantification, and early experimental confirmation (5) are all considered best practice.

### 2.6 Predictive biomarkers and personalized treatments

AI can distinguish prognostic biomarkers from predictive markers of drug response, aiding personalised treatments. The power of AI can be harnessed, if applied judiciously, across all stages of the drug discovery and development pipeline, ultimately enabling the discovery of novel and precision medicines for the treatment of various human diseases (5). The translational gap persists because most biomarker models are retrospective and lack multicenter prospective validation; standardized multi-omics datasets and harmonized clinical endpoints are required for clinical deployment (8).

### 2.7 AI in oncology and clinical trials

AI is changing drug development processes, particularly in oncology, by optimizing clinical trials, personalizing therapy, predicting toxicity, and monitoring post-market safety. AI is increasingly empowering all facets of clinical trial optimization (5, 7, 8). From patient identification and stratification to adaptive protocol design and continuous monitoring, Retrospective studies and simulations show improved recruitment and enrichment and potential reductions in required sample size; however, there are relatively few prospective randomized trials proving improved clinical outcomes driven by AI-enabled decisions (5, 8). **Table 2** summarizes representative AI applications, data sources, evidence types and validation levels (8).

**Table 2:** Representative AI applications in oncology drug development and management: tasks, exemplar methods, data sources, and evidence types.

S.No	Task	Exemplar AI method/tool	Oncology use case (illustrative)	Primary data source(s)	Evidence types in cited studies	Representative refs
1.	Discovery & design	Structure/target assessment	AlphaFold 2/3 structure prediction	Pocket/complex modelling informs target draggability	Protein structures (PDB), sequences	Structural confidence/coverage (pLDDT/lDDT); qualitative enablement
2.	Discovery & design	*De novo* molecular design	Graph-based generative models, transformers	Novel chemotypes for oncology targets	Public/enterprise chemical corpora	Retrospective hit enrichment; faster ideation cycles
3.	Discovery & design	Virtual screening/prioritization	GNN/DL rescoring; multi-task QSAR	Prioritize candidates for wet-lab validation	Assay libraries, docking outputs	Improved enrichment vs. baseline docking (context-dependent)
4.	Preclinical/translation	Mechanism-informed hypothesis generation	Knowledge graphs + link prediction	Target–compound–disease triangulation	Literature/omics/chemical DBs	Qualitative prioritization vs. manual curation
5.	Clinical trials	Eligibility screening/recruitment	EHR NLP + rules/phenomapping	Faster identification of eligible patients	EHR notes + structured fields	↑ screening efficiency; potential ↑ power
6.	Clinical trials	Control arm strategies	Synthetic controls/digital twins	Augment/replace control cohorts	Historical trials, registries, EHR	Potential sample-size reduction; faster decisions
7.	Precision therapy & dosing	Dose personalization	CURATE.AI platform	Individualized dosing in solid tumors (feasibility)	Longitudinal labs/response	Feasibility; individualized dose trajectories
8.	Resistance & combinations	Synergy prediction	Knowledge-graph embeddings; DL/GNN synergy models	Rational combination design to delay resistance	Pharmacogenomic & assay datasets	Retrospective synergy ranking; preclinical validation
9.	Toxicity & pharmacovigilance	AE extraction (EHR)	EHR-NLP pipelines	irAE monitoring in oncology	EHR clinical notes	AE frequencies concordant with trial reports
10.	Toxicity & pharmacovigilance	Signal fusion	FAERS/VigiBase + EHR-NLP	Earlier/more sensitive signal detection	Spontaneous reports + EHR	Earlier detection in fusion settings (qualitative)
11.	Supply chain & regulation	Anti-counterfeiting/traceability	Blockchain + anomaly detection	Serialization and track-and-trace	Logistics/serialization streams	Qualitative improvements in traceability

Source: Adapted and Reproduced from Artificial intelligence in oncology drug development and management: a precision medicine perspective (6**).**

### 2.8 Integrative AI-enabled oncology pipeline

Artificial intelligence has been evolving from a series of disparate tools into an integrated platform for oncology R&D. Realistically, currently, some of the most well-developed use cases involve AI applied to structured analysis of known data (e.g., radiology, pathology images, and molecular data), while other emerging use cases involve synthetic patient modeling and mechanistic target identification. The relevance of such distinctions between some and others is critical; they differentiate the clinical near-term usage from the more explorative modes that are in fact still quite far from clinical applicability and demand robust external and prospective validation (18, 25).

### 2.9 AlphaFold and oncology drug development

At the discovery end of the pipeline, AlphaFold has reshaped structural annotation and target prioritization by enabling near-complete proteome-scale structure prediction, including cancer-relevant proteins with limited experimental structures. In oncology, these models are especially useful for prioritizing mutant kinases, transcriptional regulators, and protein–protein interaction interfaces that may be difficult to resolve experimentally, thereby supporting structure-guided hypothesis generation and early lead discovery. However, AlphaFold structures are typically static and do not fully capture ligand-induced conformational change, intrinsic dynamics, or post-translationally modified states, which are central to cancer signaling and therapeutic resistance. For this reason, AlphaFold output should be treated as a starting framework that must be integrated with docking, molecular dynamics, and experimental structure determination before being used for lead optimization or validation (17, 26, 27).

### 2.10 Validation, success rates, and clinical translation

While several AI approaches report higher in silico hit rates or retrospective enrichment, there is little independent evidence that AI increases IND-to-approval success rates in oncology (5, 8). Validation studies are often limited to internal cross-validation or retrospective cohorts; prospective preclinical validation and multicenter clinical validation are rare (5). In a few selected cases where AI-supported candidates entered trials, clinical translation has been achieved. But overall measurable increases in late-stage success rates solely attributed to AI remain sparse.

### 2.11 Advantages, challenges, and recommendations

Advantages: Capacity to integrate heterogeneous data, rapid exploration of chemical space, and potential cost/time savings (28).

Challenges: Data biases and fragmentation, limited interpretability, overfitting and poor generalizability, inadequate external validation, and evolving regulatory pathways (8).

Recommendations:

- Implement comparative benchmarking and reporting of enrichment, AUC, and calibration on blinded datasets (8).
- Follow a validation hierarchy: internal cross-validation → external retrospective cohorts → prospective experimental validation → multicenter clinical evaluation.
- Report dataset provenance, preprocessing steps, model architecture, and uncertainty estimates (10).
- Engage regulators early and use pre-specified validation plans for AI-derived candidates (5).

If used wisely and validated rigorously, AI can contribute at every step of drug discovery and development, ultimately aiding the discovery of novel and precise medicines for treating a wide range of human diseases. The central translational requirement is robust, prospective validation linking AI outputs to experimental and clinical outcomes (8).

## 3 Drug repurposing with artificial intelligence

Drug repurposing using approved or clinically characterized compounds for new indications can shorten timelines and reduce costs compared with *de novo* drug discovery because repurposed compounds are often derisked with known safety and pharmacology (29). This strategy has gained renewed interest given high dropout rates, high costs, and the slow pace of new drug development (29, 30). Computational and experimental approaches powered by AI accelerate candidate identification, but technological and regulatory challenges remain (29).

### 3.1 Pandemics and repurposing rationale

Rapid outbreaks such as SARS-CoV-2 highlighted the need for fast therapeutic options and reinforced repurposing as a pragmatic approach to rapidly deploy drugs with established pharmacological profiles while focusing on pathogen host biology (31). In oncology, the analogous need is rapid translation of agents that can target shared cancer pathways or vulnerabilities across tumour types.

### 3.2 Disease-centric versus target-centric approaches

Two main drug repurposing strategies are target-centric and disease-centric approaches.

### 3.3 Disease-centric versus target-centric repurposing

Disease-centric repurposing seeks drugs that can treat different diseases that share common pathways or phenotypes, while target-centric repurposing exploits the same protein target implicated in multiple conditions.

Aspirin and other NSAIDs: Epidemiological and mechanistic data indicate lower incidence and better outcomes in some colorectal and other cancers through COX-mediated inflammation pathways, with AI-based network and pharmacoepidemiology analyses enhancing signal prioritization for specific cancer subtypes (29).

Metformin: Retrospective and mechanistic studies have demonstrated associations with reduced incidence of cancer and improved outcomes in several tumor types (breast, prostate, colorectal). ML analyses combining pharmacoepidemiology and tumor-omics have identified contexts (e.g., metabolically addicted tumors) for prospective evaluation.

Statins: Network-based repurposing and transcriptomic reversal strategies identify statins as possible candidates for specific cancers (e.g., ovarian, prostate) through their effect on the mevalonate pathway and prenylation. AI approaches have been applied to correlate tumor expression signatures with drug-induced perturbation profiles.

Disulfiram and other Repurposed Agents: Disulfiram (an alcohol-aversion drug) and its metal complexes have exhibited preclinical anticancer activity, mined from clinical data and multi-omics integration. AI approaches aided in prioritizing such candidates via PPI network analyses and chemogenomic matching.

Case Studies in Network Pharmacology: AI approaches incorporating protein–protein interaction (PPI) networks, drug–target databases, and transcriptomic signatures have identified potential repurposing candidates for specific cancer subtypes (30).

### 3.4 Target-centric mechanisms in oncology

Target-centric repurposing in cancer identifies drugs that modulate targets common to multiple diseases or tumor types (e.g., kinase inhibitors with activity across different histologies sharing the same driver mutation). AI can also map target expression, mutation frequency and pathway dependencies across tumor cohorts to nominate repurposing candidates with the highest translational potential.

### 3.5 Data integration for computational repurposing

To be repurposed effectively, they must be systematically integrated with genomics, transcriptomics, proteomics, drug–target data, chemical structure, phenotypic screens and population-level clinical data. These layers can be integrated via AI/ML methods (network algorithms, similarity-based approaches, matrix factorization, and deep learning) to identify candidate drug-disease matches and prioritize agents for experimental validation (30). Refer to **Figure 2** for a schematic overview of AI/ML Methodologies in Drug Discovery.

**Figure 2:**
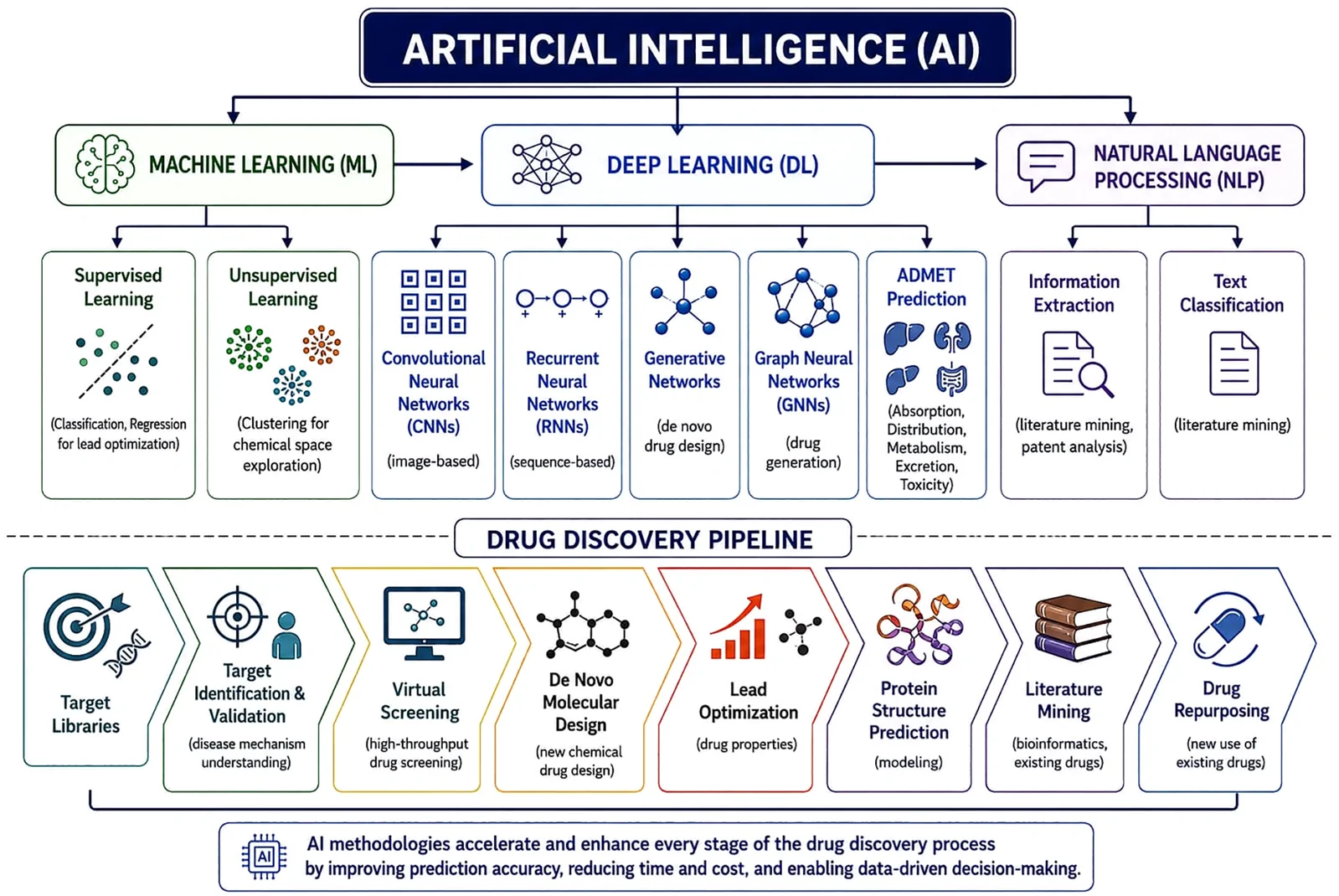
Schematic overview of AI/ML methodologies in drug discovery. Source Adapted from: Pathak et al. (10). Recreated by: Canva, Luna (https://lunaaiart.app), and BioRender (https://www.biorender.com).

### 3.6 Validation, evidence levels, and oncology translation

One major limitation of repurposing work is the disconnect between computational nomination and clinical translation. In oncology, many AI-based drug repurposing studies report in silico prioritization or *in vitro* activity, but very few progresses to *in vivo* efficacy studies or prospective clinical trials. Successful translation requires transparent reporting of methods and datasets, stepwise validation (in silico to *in vitro* to *in vivo* to early-phase clinical testing). Differences in dosing, formulation, and pharmacokinetics between original and repurposed indications and prospective tumor-specific clinical evaluations should be considered.

### 3.7 Challenges in AI-driven repurposing

Despite advances, key challenges undermine the reliability of oncology repurposing: real-world datasets are often incomplete, heterogeneous, and biased toward well-studied cancers and populations; “black-box” models can obscure decision-making rationales, reducing clinician/regulator trust; and annotation errors and publication bias can generate spurious signals. Addressing these issues requires dataset harmonization, interpretable models, uncertainty quantification, and prospective validation.

For cancer-focused repurposing, prioritize AI pipelines that integrate tumor-specific multi-omics, pharmacoepidemiology, and mechanistic assays; require stepwise experimental validation; and report evidence tiers (computational signal, *in vitro*, *in vivo*, early clinical) to support clinical prioritization. **Table 3** should summarize selected clinical trials for repurposed drugs in cancer and related proliferative conditions.

**Table 3:** Selected clinical trials for repurposed drugs in cancer and related proliferative conditions (Data derived from clinicaltrials.gov).

S.No	Trial code	Condition	Repurposed drugs	Phase	Type	Status
1.	NCT05977738	Glioblastoma Multiforme (adult), Recurrent Glioblastoma	Pitavastatin calcium	Early Phase I	Interventional	Completed
2.	NCT02770378	Glioblastoma	Temozolomide in combination with: Aprepitant/Minocycline/ Disulfiram/Celecoxib/ Sertraline/Captopril/ Itraconazole/Ritonavir/ Auranofin	Phase I, Phase II	Interventional	Completed
3.	NCT04997811	Myelodysplastic Syndromes (MDS)	Sodium Valproate/Bezafibrate/Medroxyprogesterone vs. Danazol	Phase II	Interventional	Recruiting
4.	NCT03378297	Ovarian cancer	Metformin/Acetylsalicylic acid/Olaparib/Letrozole	Early Phase I	Interventional	Completed
5.	NCT02101008	Melanoma	Disulfiram and Chelated zinc	Phase II	Interventional	Completed
6.	NCT01220973	Prostate cancer	Atorvastatin calcium and Celecoxib	Phase II	Interventional	Completed
7.	NCT01101438	Breast cancer	Metformin	Phase III	Interventional	Completed
8.	NCT03109873	Head and Neck cancer	Metformin	Early Phase I	Interventional	Completed
9.	NCT00582660	Colorectal Adenoma, Colorectal Carcinoma	Celecoxib	Phase II	Interventional	Completed
10.	NCT02896907	Pancreatic Adenocarcinoma, Recurrent Pancreatic Carcinoma, Stage III Pancreatic Cancer, Stage IV Pancreatic Cancer, Unresectable Pancreatic Carcinoma	Chemotherapy (Oxaliplatin/Irinotecan Hydrochloride/Leucovorin Calcium/Fluorouracil) in combination with ascorbic acid	Early Phase I	Interventional	Completed
11.	NCT00462280	Precancerous Condition, Stage 0, Stage I, Stage II Melanoma	Lovastatin	Phase II	Interventional	Completed
12.	NCT00094445	Pancreatic Neoplasms, Adenocarcinoma	Curcumin	Phase II	Interventional	Completed
13.	NCT02227316	Symptomatic Uterine Fibroids and Adenomyosis	Ibuprofen vs. Acetaminophen	Phase IV	Interventional	Completed
14.	NCT02913612	Infantile Hemangioma	Timolol	Phase II	Interventional	Completed
15.	NCT00290758	Breast cancer	Genistein	Phase II	Interventional	Completed
16.	NCT01844583	Solid Tumors, Lymphoma	Alisertib in combination with Esomeprazole or Rifampin	Phase I	Interventional	Completed

Trials that have been terminated are not included;

Source: Adapted and reproduced from Keramida et al. (32) on AI-driven drug repurposing applications and challenges.

## 4 Artificial intelligence in precision oncology

### 4.1 Precision medicine and clinical genomics

Precision medicine refines disease definitions using genomic and other molecular data to enable targeted therapies for well-defined patient subgroups. Prominent examples include cystic fibrosis and cancer (33). Clinical genomics exists at the intersection of sequencing-led discovery genetics in population cohorts and historically low-throughput approaches to genetic diagnosis in patients. As a result of the different aims of these two endeavors, the technologies and algorithms developed for discovery genomics need to be optimized before their application to clinical medicine (33). Precision oncology is an innovative approach to cancer care in which diagnosis, prognosis, and treatment are informed by the individual patient’s genetic and molecular profile. The successful translation of precision oncology into daily practice requires prospective testing and regulatory review rather than relying solely on biological plausibility (15).

### 4.2 Omics data growth and AI opportunities

Rapid advances in high-throughput omics technologies have generated massive, complex patient datasets, which in turn have prompted the development of novel computational infrastructures, platforms, and tools to store, retrieve, and analyze the data efficiently. Artificial intelligence (AI), and particularly its subfield of machine learning, is ideal for deciphering patterns in large datasets and offers unique opportunities for advancing precision oncology (34). Artificial intelligence (AI) in cancer medical imaging involves Deep learning algorithms that have been used as a powerful tool in healthcare for medical imaging, used to monitor disease diagnosis, aid surgical procedures, and manage the disease. Precision oncology research also benefits from a variety of alternative ML approaches for supervised and unsupervised pattern analysis in datasets originating from multiple sources, including tumor-derived omics profiles. A measure of how useful these models are should be taken, including whether they enhance diagnostic reliability and treatment decisions, and whether they improve survival rates, reduce toxicity, and lower costs compared with more standard options. Just showing good performance in retrospective studies may not be adequate, and more comprehensive prospective studies would be required to ensure that this work performs for all patient groups and healthcare settings. Disparities in healthcare can be a problem, especially given that many models are based on incomplete or poorly defined datasets and consequently do not work as effectively in less well-represented populations.

### 4.3 AI in radiology and imaging modalities

AI has significantly enhanced image analysis across X-ray, ultrasound, CT, MRI, PET, and digital pathology. It increases speed, sensitivity, and reproducibility (34). Deep learning radiomics extracts quantitative imaging features that correlate with molecular phenotypes and outcomes; these radiomic signatures can aid in non-invasive tumor characterization and monitoring over time. AI in radiology supports early detection, lesion segmentation, and response assessment, which reduces variability between readers and enables scalable screening workflows (34, 35). For imaging AI to be useful in clinics, a comprehensive validation across scanners, institutions, and patient populations is necessary, since the performance can fall when the models are used in a different environment from that in which they were developed. Integration into workflow within the PACS/RIS environment, together with supervision from radiologists and well-defined clinical goals, is important (36, 37). Explainable AI techniques can provide useful context in radiology to justify why certain areas of interest have been highlighted by the model and indicate how it would be graded (38, 39).

### 4.4 Radiogenomics and multimodal integration

Radiogenomics and liquid biopsy analytics indicate how cancer could go from single samples to non-invasive longitudinal follow-up as images are transformed by machine learning to link imaging phenocryptotypes with genomic/transcriptomic profiles. This enables the inference of mutation status, signaling pathway activity, and treatment response from image data alone. However, they remain subject to site-specific protocols and lack well-defined annotated data, making them highly useful but needing to be verified on broad datasets with clearly defined endpoints (40, 41). Radiogenomics connects imaging phenotypes with underlying genomics and transcriptomics, allowing for non-invasive inference of mutation status, expression patterns, and pathway activity. AI models that learn from imaging and molecular data together (multimodal models) can predict clinically relevant alterations (e.g., EGFR, IDH) and therapy responses without needing tissue prebiopsy. Recent developments in large multimodal architectures improve the merging of different inputs and support cross-modal retrieval and prediction. Even though promising radiogenomic models exist, many studies remain at a retrospective or single-institution level, suggesting their full clinical utility should be based on external validation, calibration, and demonstrable treatment-changing decisions or benefit to the patient. All these types of imaging analyses need to demonstrate their performance on a wider population using more diversified imaging protocols (37).

### 4.5 Digital pathology and feature discovery

Digital pathology allows for high-resolution image analysis at scale, reducing variability. Algorithms can discover morphologic biomarkers linked to diagnosis, prognosis, and treatment response (13). Tools like SMILY (similar image search for histopathology) demonstrate the unsupervised retrieval of morphologically similar regions from large unlabeled databases (42). Highly validated models like LYNA achieve near-human performance for specific tasks, such as detecting metastasis, though external validation and workflow integration remain crucial. These can be seen from translational perspectives as valuable enablers; however, consistent external validation and routine use require careful collaboration with laboratory testing systems and the overall delivery process of reports. Cost effectiveness and time can also be factors that could affect the uptake and development of a digital platform; even though digital pathology can now complement histopathology, many stages of the process will need more robust training and workflow development before it can be widely implemented (43).

### 4.6 Foundation models and large multimodal models in pathology

Foundation models are likely the best developed so far in pathology, with performance demonstrated across multiple scanners, institutions, and variations in staining, because of extensive pre-training. Their functions are widely demonstrated for diagnosis, grading, prognosticating biomarkers, and outcomes classification. They can also incorporate whole slide images along with clinical information and other data, being part of a multimodal combination. Foundation models can assist by creating links between data and assisting, for example, with clinical decision making or with designing new studies and standardizing datasets among institutions, which differ from systems that may rely on deep mechanistic models like AlphaFold that could only potentially be validated as part of routine care (44). Despite the potential, they are primarily based on retrospective data, and there is only limited evidence of clinically useful results. Validation across institutions in a well-controlled and calibrated study will also need to show its superiority in diagnostic accuracy, clinical outcomes, and patient treatment choice. This will depend on how they receive approval by regulations, calibration protocols, etc and how their performance reflects that in actual practices (37, 45).

### 4.7 AI-assisted clinical decision support systems

Clinical support systems such as IBM Watson for Oncology help to summarize the literature and give advice on cancer treatment based on a variety of tumors (46–49). However, they have shown variability compared to the multidisciplinary tumor boards, and are not very clear, so these tools will have to go through a formal certification process to make sure that the way they are integrated into the workflow is the ones that improve their quality. They can be useful in the existing guidelines, but again there is no proof that these devices don’t need supervision by the physicians, and therefore these are assistive rather than autonomous decision devices and their performance must be continuously supervised and adapted to follow guidelines and verify whether they increase costs or decrease the quality of care, given the importance to obtain authorization for the devices before deployment (50, 51).

### 4.8 Liquid biopsy, ctDNA analytics, and AI

AI for liquid biopsy is improving but is mostly applied in the ctDNA analysis, which is able to detect the lowest levels of variations and reconstruct DNA fragments and methylation patterns, identify clonal populations, and track minimal residual disease. Given that the ctDNA is collected in cycles and in small quantities, the use of artificial intelligence is more interesting for disease tracking and identification of minimal recurrence and patient treatment follow-up. It’s probably one of the technologies described with greater translation interest, because it helps solve the critical clinical problem of tracking patients regularly, invasively. The question of its application in real-life practice revolves around its standardization and how the analysis may help treat patients; the limitation is not the concept, but how it will be applied (52–56). AI methods have improved the sensitivity and specificity of liquid biopsy interpretation. This includes analyzing circulating tumor DNA (ctDNA) for variant calling, fragmentomics, methylation profiling, and clonal deconvolution. Machine learning enhances signal detection in samples with low tumor fractions and integrates longitudinal ctDNA changes to track minimal residual disease (MRD) and early relapse. These methods allow for less invasive molecular monitoring and may aid in early intervention. Future research needs to develop prospective methods to show whether AI-supported liquid biopsy improves identification and follow-up of early recidivation, monitoring treatments or even directing medical actions towards better treatment strategies for patients compared with current tests. Further important aspects that need to be addressed are how to optimize assay harmonization and define standards to have reliable results, taking care of the differences in the healthcare systems in terms of accessibility of testing, equity of access, and costs (56).

### 4.9 Single-cell sequencing and AI pipelines

Single-cell RNA, DNA, and proteomic technologies create high-dimensional datasets that capture intra-tumor variation and the tumor microenvironment. AI pipelines, including graph neural networks, manifold learning, and clustering with batch correction, identify rare subpopulations, infer lineage and cell-cell interactions, and predict resistance to therapy mechanisms. Combining single-cell data with spatial transcriptomics and imaging further refines the study of tumor biology. Although these processes can offer valuable insights to facilitate discoveries, their clinical translation will be limited by their processing steps, variability in batch effects, the lack of a clinical landmark for diagnosis, and the translation to a patient’s clinical treatment plan. It is vital to establish a correlation with individual therapy choice, classification of patients, and to develop an overall framework for future prospective clinical studies.

### 4.10 Clinical genomics, targeted sequencing, and trade-offs

Targeted sequencing panels are the standard in clinical workflows due to their cost and quick turnaround. They capture actionable genes (BRCA1/2, TP53, PTEN, KRAS, BRAF, ERBB2) with high sensitivity but may miss large rearrangements and new loci (22, 57–60). Whole-exome and whole-genome sequencing (WES/WGS) can address these gaps, although they entail higher costs and computational demands (61). AI can simplify analysis by automating variant annotation, prioritizing clinically relevant changes, and flagging structural events, thereby speeding up interpretation pipelines. For example, AI is becoming increasingly essential in clinical genomics, where its ability to reduce interpretation times, prioritize candidate variations and provide actionable reports is very helpful for the precise management of patients, but we will need regulations and reimbursement structures before these devices can be considered for broad adoption, and interoperability with laboratory information systems.

### 4.11 Digital twins and patient avatars

Digital twins add an exciting, more advanced layer to the potential use of AI for cancer patient treatment, creating patient-specific computational models derived from multimodal data (clinical, genomic, imaging, and treatment). These avatars, known as digital twins, promise to predict patient evolution and suggest specific treatments by modeling in silico alternative treatments and/or sequences of therapies, which could be further enhanced by using multimodal foundation models and mechanism knowledge graphs (62).

This offers substantial potential for individualized treatment sequence predictions, toxicity prognosis and adaptive monitoring. However, these models still suffer from significant gaps in the completeness of data capture and uncertainty in prediction due to model imperfections. Furthermore, clinical validation against the ‘true’ course of disease is notoriously difficult for these systems. Therefore, they should be regarded as translation research platforms or investigative tools at this stage, and not as standard clinical decision-making tools. Key requirements for translation will be the need for transparency regarding uncertainty, as well as external validation and clear frameworks for the ethical application of these tools, particularly concerning treatment recommendations. Their most probable function is for exploration and hypothesis generation and not for prescription until their reliability can be firmly established in prospective trials demonstrating clinical benefit, cost-effectiveness, and addressing fairness through careful validation and consideration of any known data gaps or biases (62).

### 4.12 Performance, validation, and implementation challenge

Though AI models demonstrate strong performance in retrospective tasks, translation to real-world use requires external validation across multiple centers, prospective trials, and attention to shifts in datasets, batch effects, and population biases (63, 64). Foundational and multimodal models enhance transferability but require comparison against diverse clinical data (65, 66). Explainability, uncertainty quantification, and clear reporting standards are vital for gaining clinicians’ trust and regulators’ acceptance (67, 68). Prospective validation studies are needed to determine whether AI improves hard clinical outcomes such as survival, toxicity reduction, time to diagnosis, or decision accuracy (15, 45). Further prospective validation is needed to assess whether AI offers benefits on hard clinical endpoints such as improved survival, decreased toxicity, reduced delay to diagnosis, and increased diagnostic accuracy (15, 45). Because retrospective performance may overstate clinical value, we need real-world data generated in multi-institutional deployment (37). Discussion of regulatory approval paths may be warranted, given that ultimate clinical deployment depends upon classification of the system as software as a medical device, decision-support software, or fully automated decision support (69). Laboratory, PACs, EMR, and tumor board integration will all play a role. Cost-effectiveness and reimbursement will influence the uptake of AI beyond specialized academic centers (45). Clinically, we view explainable AI as an indispensable prerequisite to clinical trust, and this becomes ever more salient in oncology decision-making, where clinicians must often be able to reason about why a prediction was made (39). AI, particularly when utilizing foundation and large multimodal models, radiogenomics, single-cell pipelines, liquid biopsy analytics, and digital twin frameworks (34, 70, 71), has the potential to revolutionize precision oncology. Achieving clinical benefits relies on thorough validation, prospective evaluation, and smooth integration into clinical workflows under clinician oversight (63, 72, 73). Achieving clinical benefit will require prospective validation, real-world evidence, regulatory alignment, explainable outputs, workflow integration, and attention to equity and cost-effectiveness, with clinician oversight remaining central to safe deployment.” That wording is well aligned with the translation-focused evidence base across imaging AI, genomics, pathology, and decision-support systems (37, 45, 51).

## 5 Artificial intelligence in multi-omics data integration

Multi-omics integrates diverse biological data types, including genomics, transcriptomics, proteomics, metabolomics, and epigenomics, to build a comprehensive understanding of human health and disease (12). Integration and analysis of multi-omics data help us derive useful information about cellular functions. It provides us with a holistic view of the complex biological system by integrating important information from various layers of omics data (74, 75).

### 5.1 Rationale for a multi-layer study

Complex biological phenomena emerge from interactions across multiple layers of biological features; therefore, joint analysis outperforms single-layer studies in capturing a more complete description of these phenomena (75). There is a plethora of publicly available databases (TCGA, CPTAC, ICGC, METABRIC, etc.) that provide multi-omics datasets of patients. **Table 4** provides a list of publicly available multi-omics cancer datasets.

**Table 4:** List of publicly available multi-omics cancer datasets.

S.No	Dataset	Description
1.	BRCA (Breast Cancer), STAD (Stomach Cancer), COAD (Colon Cancer) - TCGA (The Cancer Genome Atlas)	The BRCA, STAD, and COAD datasets are vital to TCGA, encompassing genetic and clinical data on breast, stomach, and colon adenocarcinomas. These resources, curated by the NCI and NHGRI, amalgamate genetic mutations, gene expression profiles, and treatment responses.
2.	ICGC (International Cancer Genomics Consortium)	The International Cancer Genome Consortium (ICGC) dataset is a collaborative effort among researchers worldwide to collect and analyze genomic data from various types of cancer. It includes information on genetic mutations, gene expression patterns, epigenetic modifications, and clinical data from thousands of cancer patients.
3.	METABRIC (Molecular Taxonomy of Breast Cancer International Consortium)	The METABRIC dataset is a comprehensive genomic and clinical resource focusing on breast cancer. It combines data from two large-scale studies: the Molecular Taxonomy of Breast Cancer International Consortium (METABRIC) and The Cancer Genome Atlas (TCGA). This dataset includes information on genetic mutations, gene expression profiles, clinical characteristics, and patient outcomes from a large cohort of breast cancer patients.
4.	CCLE (Cancer Cell Line Encyclopedia)	The Cancer Cell Line Encyclopedia (CCLE) dataset comprises genomic and pharmacological data from a diverse collection of cancer cell lines. It includes information on gene expression, genetic mutations, and drug sensitivity profiles across various cancer types. The CCLE dataset serves as a valuable resource for understanding cancer biology, identifying novel therapeutic targets, and predicting drug responses.
5.	TARGET (Therapeutically Applicable Research to Generate Effective Treatments)	The Therapeutically Applicable Research to Generate Effective Treatments (TARGET) dataset is a comprehensive resource focusing on pediatric cancers. It includes genomic, transcriptomic, and clinical data from large cohorts of pediatric cancer patients. TARGET dataset aims to uncover the genetic basis of pediatric cancers, identify therapeutic targets, and improve treatment outcomes for children with cancer.
6.	OmicsDI (Omics Discovery Index)	The OmicsDI dataset is a centralized platform that integrates various omics data, including genomics, proteomics, metabolomics, and transcriptomics, from different repositories worldwide. It provides a unified interface for accessing and analyzing multi-omics data, facilitating cross-disciplinary research and data-driven discoveries.
7.	OmicsDI (Omics Discovery Index)	The OmicsDI dataset is a centralized platform that integrates various omics data, including genomics, proteomics, metabolomics, and transcriptomics, from different repositories worldwide. It provides a unified interface for accessing and analyzing multi-omics data, facilitating cross-disciplinary research and data-driven discoveries.
8.	Burdenko-GBM (Glioblastoma)	The Burdenko-GBM dataset is a specialized resource focusing on glioblastoma multiforme (GBM), the most aggressive form of brain cancer. It comprises genomic, transcriptomic, and clinical data from patients diagnosed with GBM. The dataset aims to elucidate the molecular mechanisms driving GBM development and progression, identify potential therapeutic targets, and improve patient outcomes.
9.	SARC Clinical Data Repository	The SARC dataset focuses on sarcomas, a diverse group of rare cancers arising from connective tissues. It encompasses genomic, transcriptomic, and clinical data from patients diagnosed with various subtypes of sarcoma. The dataset aims to unravel the molecular landscape of sarcomas, identify biomarkers for diagnosis and prognosis, and discover potential therapeutic targets.

Source: Adapted and reproduced from Acharya & Mukhopadhyay (75).

### 5.2 Challenges and AI benefits

Multi-omics data are high-dimensional, heterogeneous, complex, and often sparsely sampled. Artificial Intelligence (AI), particularly deep learning and neural networks, has emerged as a powerful tool to overcome these limitations by enabling advanced data integration, biomarker discovery, and personalized treatment strategies (12). AI also supports feature selection, network reconstruction and uncertainty estimation, all crucial for clinical translation. Deep learning is important for combining multi-omics data because it can learn from datasets that are big, complicated, and different from each other (12). The growing availability of high-throughput sequencing and mass spectrometry technologies has accelerated multiomics research, enabling a more detailed exploration of cellular and molecular mechanisms underlying diseases (76).

### 5.3 Network-based reasoning

Graph neural networks (GNNs) serve as an intuitive architecture to conceptualize cancer as an interconnected system rather than just an isolated set of genes. A graph in an oncology context might represent genes, proteins, cells, metabolites, or drugs as nodes, linked by biological associations as edges. Many biologically meaningful networks exist (e.g., protein– protein interaction networks, gene regulatory circuits, signaling cascades, or spatial/single-cell cellular neighborhoods). Graph networks in oncology find their most compelling uses in the areas of targeting priority e.g. Identifying driver proteins from network information, multiomics integration, integrating transcriptomic and proteomic data on the same graphs, drug repositioning (identifying new therapeutic indications of existing drugs based on similarity of network context to known indications), and analysis of single-cell data, spatial transcriptomics, or even tissue structural information (54). The value of graph networks in oncology lies in circumstances when the question hinges on network context (e.g., identifying pathway-level vulnerabilities in the network or modeling interactions between different cells), as they extend our understanding beyond the single gene/protein/cell. While currently they are less mature as standalone clinical instruments, they are of substantial interest for use as integrative tools. Many types of biologically related information can be modelled as graphs to bring together diverse inputs, such as molecular information, into a unified biological narrative that makes other, disparate, AI components more sensible (e.g., radiogenomics output can be integrated into GNNs to make sense of tumor spatial context). Key uses of GNNs involve:

Drug-target prioritization: GNNs transmit functional signals through protein-protein interaction networks to identify targets or candidates for repurposing.

Multi-omics fusion: GNNs combine omics features at nodes (like gene expression and mutation status) to learn pathway-level representations that can predict subtypes or drug responses (77).

Spatial and single-cell analysis: GNNs capture neighborhood relationships in spatial transcriptomics and infer cell-cell interaction networks linked to prognosis (13, 42).

### 5.4 Multi-modal transformers and foundation models

Multimodal transformer models unify the treatment of multiple types of clinical data, including genomic sequences, expression matrices, histopathology whole slide images, and clinical text notes, within a shared latent space to enable cross-modal retrieval and zero-shot transfer (78). Foundation models are pre-trained on abundant archives of biological data and medical images to provide a strong feature extraction engine that can then be trained for more specific downstream tasks using a small dataset with either traditional fine-tuning or prompting strategies. Foundation models are an effective technique for rapidly developing many predictive diagnostic tools, especially in the fields of imaging and pathology, and they generalize well but are currently more brittle to calibration issues than required for immediate safe clinical deployment (79–81).

### 5.5 Federated learning and privacy preservation

Federated learning allows collaboration on training a single, powerful model among multiple institutions without raw patient data ever leaving individual data centers. Federated learning has great potential for further fine-tuning large foundation models for specific cancer datasets and building generalizable multi-institutional models for tasks like survival prediction. Hurdles remain in areas like defining harmonized feature definitions and data schemas, designing robust server-side aggregation and client-side data selection protocols, and preventing malicious attacks. Protocols that incorporate robust security and differential privacy can address some of these limitations (79, 80). The current most successful implementations often rely heavily on rigorous harmonization, iteratively validating against test institutions and maintaining strict vigilance for drift over time.

### 5.6 Clinical applications and evidence level

Molecular subtyping: Integrated transcriptome–proteome models have produced prognostic molecular subtypes in oncology with retrospective validation across cohorts (80).

Drug sensitivity prediction: Developing multi-omics models capable of predicting cellular response to targeted drugs is a burgeoning area that could eventually facilitate personalized treatments, and some multi-omics approaches have entered small-scale, *in vitro* translational or early-stage clinical studies (80).

Spatial biomarkers: Combining genomics information with spatial resolution through spatial transcriptomics and with pathology, Whole Slide Imaging has recently yielded a wealth of biological insights into tumor microenvironment structures predicting response to immunotherapy. While extremely interesting, much of this work is primarily in retrospective or very small prospective cohorts and awaits further validation in large, multi-center, randomized clinical trials. The majority of work in molecular and imaging AI has demonstrated very strong retrospective performance, with only a few small-scale prospective studies conducted to date (82).

Evidence level summary: While multiple studies show strong retrospective performance and small prospective validations, the field lacks extensive large, multicenter prospective trials with pre-specified endpoints and prespecified calibration testing for clinical deployment.

### 5.7 Methods and pipeline components

Fusion strategies: Common multimodal fusion approaches include early fusion (feature concatenation), intermediate fusion (modality-specific encoders followed by joint modeling), and late fusion (ensembles or stacked predictors); empirical comparisons and architectural taxonomies in computational imaging highlight trade-offs between performance and computational cost.

Preprocessing: Robust pipelines use modality-specific normalization, batch-effect correction (e.g., ComBat variants for omics), and principled handling of missing modalities (imputation, modality dropout, or conditional encoders) to reduce confounding and improve generalizability.

Representation learning: Autoencoders, contrastive/self-supervised learning, and multimodal transformers are effective for learning shared embeddings from unlabeled biomedical data, with recent reviews documenting gains from large-scale self-supervision in medical imaging and pathology.

Downstream tasks: Typical tasks include survival prediction, classification, causal inference, and treatment-response modeling; uncertainty quantification (e.g., Bayesian ensembling, deep ensembles, or conformal prediction) is critical for clinical risk stratification.

Interpretability tools: Feature-level attribution (SHAP, Integrated Gradients) and image saliency (Grad-CAM variants) remain useful for model inspection; graph-based attribution methods are emerging for spatial and interaction models.

### 5.8 Best practices and recommendations

Robust benchmarking should use public, blinded datasets and include external validation on independent cohorts, with institution-level holdouts and temporal splits used to evaluate model robustness and generalizability. In parallel, data harmonization should standardize preprocessing pipelines and metadata to mitigate batch effects and enable reproducible cross-study comparisons (83).

Uncertainty quantification and calibration are essential, particularly for low tumor-fraction samples and rare molecular subtypes where the cost of misclassification is high. When data pooling is not feasible, privacy-preserving strategies such as federated learning and differential privacy should be preferred, together with threat modeling and robustness checks against adversarial clients. High-performing models should also undergo mechanistic validation through pathway analysis, perturbation experiments, and orthogonal assays to establish biological plausibility and strengthen clinical trust.

### 5.9 How these technologies interact

Multi-modal transformers and foundation models provide shared representations that simplify federated adaptation and cross-site transfer by decoupling feature extraction from task-specific supervision. Federated learning enables these large pretrained representations to be further adapted across institutions without data pooling, though success depends on harmonized inputs and robust aggregation. Combining multimodal representation learning with federated fine-tuning offers a realistic path toward scalable, privacy-preserving clinical models when accompanied by coordinated validation.

### 5.10 Maturity and clinical validation ranking

Most mature: Foundation models for medical imaging (radiology/pathology) and vision-language models have well-developed toolchains and many retrospective validations and are closest to clinical translation in diagnostic workflows.

Intermediate maturity: Multimodal fusion models that integrate imaging with genomics/clinical data show strong retrospective performance and pilot prospective studies but need larger multicenter prospective trials for regulatory confidence.

Largely experimental: End-to-end multimodal models that directly recommend individualized therapies (closed-loop treatment suggestion) and federated causal-inference pipelines remain largely experimental and require rigorous interventional validation.

### 5.11 Synthesis across technologies

AlphaFold and GNNs primarily support mechanism discovery and target prioritization; foundation models and radiogenomics support phenotype interpretation and biomarker prediction; ctDNA analytics support longitudinal monitoring, and digital twins aim to integrate all these layers into individualized simulation and treatment planning. The most clinically mature methods are currently pathology foundation models, radiogenomics, and AI-assisted liquid biopsy interpretation, whereas AlphaFold-guided target discovery, GNN-based network modeling, and digital twins are still largely discovery-oriented or preclinical.

A useful way to frame the field is therefore by translational readiness rather than by algorithm class. Methods that analyze routinely generated clinical data are closer to implementation, while methods that attempt to simulate hidden biology or future trajectories require stronger evidence, better calibration, and prospective multicenter validation.

### 5.12 Validation and clinical utility

Validation in oncology AI should be discussed at three different levels: technical performance, clinical utility, and real-world implementation. Technical success does not necessarily translate into patient benefit, as shown by radiotherapy targeting studies in which geometric segmentation metrics did not always correlate with clinical usefulness, underscoring the need for outcome-oriented evaluation rather than benchmark-only reporting (83–85). Similarly, real-world adoption studies in automated radiotherapy planning indicate that AI can generate clinically deliverable plans, but performance still depends on local workflows, human oversight, and disease-specific implementation conditions (84). A concrete example of successful validation is the growing class of oncology-specific AI-enabled devices that have received regulatory clearance or authorization, particularly for imaging and workflow support applications. These systems demonstrate that AI can achieve enough reproducibility and operational value for controlled deployment, but their approval also highlights a key boundary: many authorized tools support pattern recognition or quantification rather than autonomous diagnosis or treatment selection. In parallel, recent oncology-focused validation work has shown that autonomous or agentic AI systems can be tested in realistic clinical scenarios, but these studies remain controlled demonstrations rather than broad clinical adoption (9, 21) In parallel, recent oncology-focused validation work has shown that autonomous or agentic AI systems can be tested in realistic clinical scenarios, but these studies remain controlled demonstrations rather than evidence of broad clinical adoption (21).

The most established clinical applications are in image-based oncology workflows, especially radiology, radiotherapy planning, and pathology assistance. In these areas, AI has already shown value in segmentation, contouring support, and automated measurement, and several systems have been cleared for clinical use or integrated into vendor-supported workflows (9, 86, 87). These applications are comparatively mature because they operate on well-defined inputs, can be benchmarked against human experts, and often fit into existing clinical decision pathways without requiring major changes to oncology care. Even here, however, clinical success should not be overstated. Performance gains in retrospective datasets may overestimate real-world benefit, and some studies have shown that model outputs vary when applied across institutions, expert styles, or imaging protocols. Therefore, this group should be described as established but still implementation-sensitive, meaning the technology is usable in practice yet remains dependent on local validation, calibration, and oversight (9, 86, 87).

### 5.13 Emerging translational technologies

Foundation models, large multimodal models, digital twins, and AI-assisted treatment planning belong in a different category: they are highly promising, but most remain translational rather than fully clinical. Foundation models in pathology and multimodal oncology can improve feature extraction, cross-modal integration, and prediction from limited labeled data, but current evidence is still dominated by retrospective or benchmark-based studies (44, 88). Likewise, large multimodal models can combine pathology, genomics, imaging, and clinical text into a shared representation, yet their clinical impact will depend on whether they generalize across institutions and maintain calibration under distribution shift (88).

AI-assisted treatment planning also falls into this emerging category. Studies in automated breast cancer planning and multicenter radiotherapy planning suggest that AI can create plans that are clinically acceptable or close to clinical standards, but these studies still require workflow integration, human editing, and prospective outcome evaluation (85, 89). In the same way, digital twins should be presented as a research frontier rather than a clinically established tool, because their promise lies in personalized simulation, not yet in validated patient management. This distinction is important because it prevents the manuscript from implying that technical feasibility already equals clinical readiness.

### 5.14 Future directions

Future research should focus on prospective multicenter validation, model calibration, and patient-centered endpoints rather than on algorithmic novelty alone. For multimodal transformers and foundation models, the key question is not whether they can combine heterogeneous data, but whether they improve treatment decisions, survival, toxicity prediction, or care efficiency in real oncology settings. For federated learning, the most pressing need is to demonstrate that privacy-preserving training can support robust cross-site generalization without introducing hidden bias or unstable aggregation behavior (90–92).

A useful way to present the field is therefore to organize it into three tiers: established clinical applications, emerging translational technologies, and future research directions. Established applications include oncology imaging support and radiotherapy planning systems with regulatory clearance or real-world implementation evidence. Emerging translational technologies include pathology foundation models, multimodal transformers, federated learning, and AI-assisted treatment planning. Future directions include digital twins, autonomous oncology agents, and fully integrated multimodal decision systems that still require substantial prospective validation before they can be considered routine clinical tools.

### 5.15 Advantages over conventional methods

Conventional statistical methods encounter difficulties with the high-dimensional characteristics of multi-omics datasets, whereas AI-driven models proficiently capture nonlinear relationships and intricate interactions among various omics layers (77). In addition to feature selection, AI has enhanced multi-omics network analysis, enabling the construction of biological interaction maps that reveal key molecular interactions. Graph neural networks (GNNs), a powerful AI technique, have been applied to model complex relationships between genes, proteins, and metabolites, uncovering novel therapeutic targets. Random forests, on the other hand, have been instrumental in biomarker discovery, identifying the most relevant features from multi-omics datasets (77). Furthermore, natural language processing (NLP) and AI-driven knowledge graphs have been leveraged to integrate multi-omics data with biomedical literature, improving the interpretation of biological findings in the context of existing research (93). AI-driven NLP tools have facilitated the extraction of disease-related genetic interactions from extensive biomedical databases, thereby advancing the identification of novel disease mechanisms (94). Because the field spans both mature clinical tools and exploratory computational frameworks, it is useful to classify AI technologies in oncology by translational readiness rather than by algorithm type alone. **Table 5** summarizes the development stage, validation status, clinical readiness, and principal limitations of each approach, highlighting that pathology foundation models and liquid biopsy analytics are closest to clinical use, whereas AlphaFold-guided discovery, graph neural networks, and digital twins remain more exploratory.

**Table 5:** Summarises the development stage, validation status, clinical readiness, and principal limitations of various AI technology approaches.

S.No	Technology	Development stage	Validation level	Clinical readiness	Key limitations
1.	AlphaFold- guided structural modeling	Advanced preclinical	Moderate; strong benchmark performance, limited oncology-specific prospective validation	Low to moderate	Static conformations, limited ligand-bound states, poor representation of protein dynamics and post-translational modifications
2.	Foundation models in pathology	Early clinical translation	Moderate to high; supported by retrospective multi-site studies and benchmark datasets	Moderate to high	Domain shift across scanners and stains, calibration issues, interpretability, need for external prospective validation
3.	Liquid biopsy AI for ctDNA analysis	Early clinical translation	Moderate; increasing evidence for MRD, relapse prediction, and longitudinal monitoring	Moderate	Low tumour fraction, assay heterogeneity, standardization challenges, limited prospective outcome data
4.	Radiogenomics and multimodal imaging models	Early translational	Moderate; mainly retrospective and single-/multi-centre validation	Moderate	Scanner variability, heterogeneity in imaging protocols, limited generalizability, annotation burden
5.	Graph neural networks	Preclinical to early translational	Moderate in benchmark and retrospective datasets	Low to moderate	Limited prospective validation, explainability concerns, integration complexity across heterogeneous omics layers
6.	Digital twins and patient avatars	Experimental	Low to moderate; mostly conceptual or pilot-level validation	Low	Incomplete longitudinal data, uncertainty in counterfactual prediction, ethical and regulatory concerns

To complement the narrative synthesis, it is useful to classify AI technologies in oncology according to translational readiness rather than algorithmic novelty alone. As summarized in **Table 5**, pathology foundation models and liquid biopsy analytics are currently the closest to clinical implementation, whereas AlphaFold-guided discovery, graph neural networks, and digital twins remain more exploratory and require stronger prospective validation. This framework clarifies how the different technologies contribute at distinct points in the oncology pipeline and helps distinguish mature clinical tools from promising but still experimental approaches.

## 6 Artificial intelligence in predicting drug toxicity and ADMET

Toxicity measures how harmful a substance can be to living things, like plants, animals, bacteria, and people (19). Given the potential health threats associated with chemical exposure, thoroughly assessing these substances in the environment is essential. Regulatory frameworks usually rely on hazard identification, dose-response characterization, exposure, and risk (95). These needs motivate rigorous, well-validated computational approaches that can reliably triage compounds before expensive experimental testing.

### 6.1 Rise of computational approaches

The rise of computational methods, along with more available experimental data, has enabled cost-effective and time-efficient strategies for early drug discovery (64, 96). Integrating AI-based toxicity prediction models into virtual screening processes filters out compounds likely to show toxicity before *in vitro* testing, improving downstream success rates and making lead optimization more efficient (63, 64, 96). Refer to **Figure 3** for Integration of AI-based toxicity prediction and Workflow of model development.

**Figure 3:**
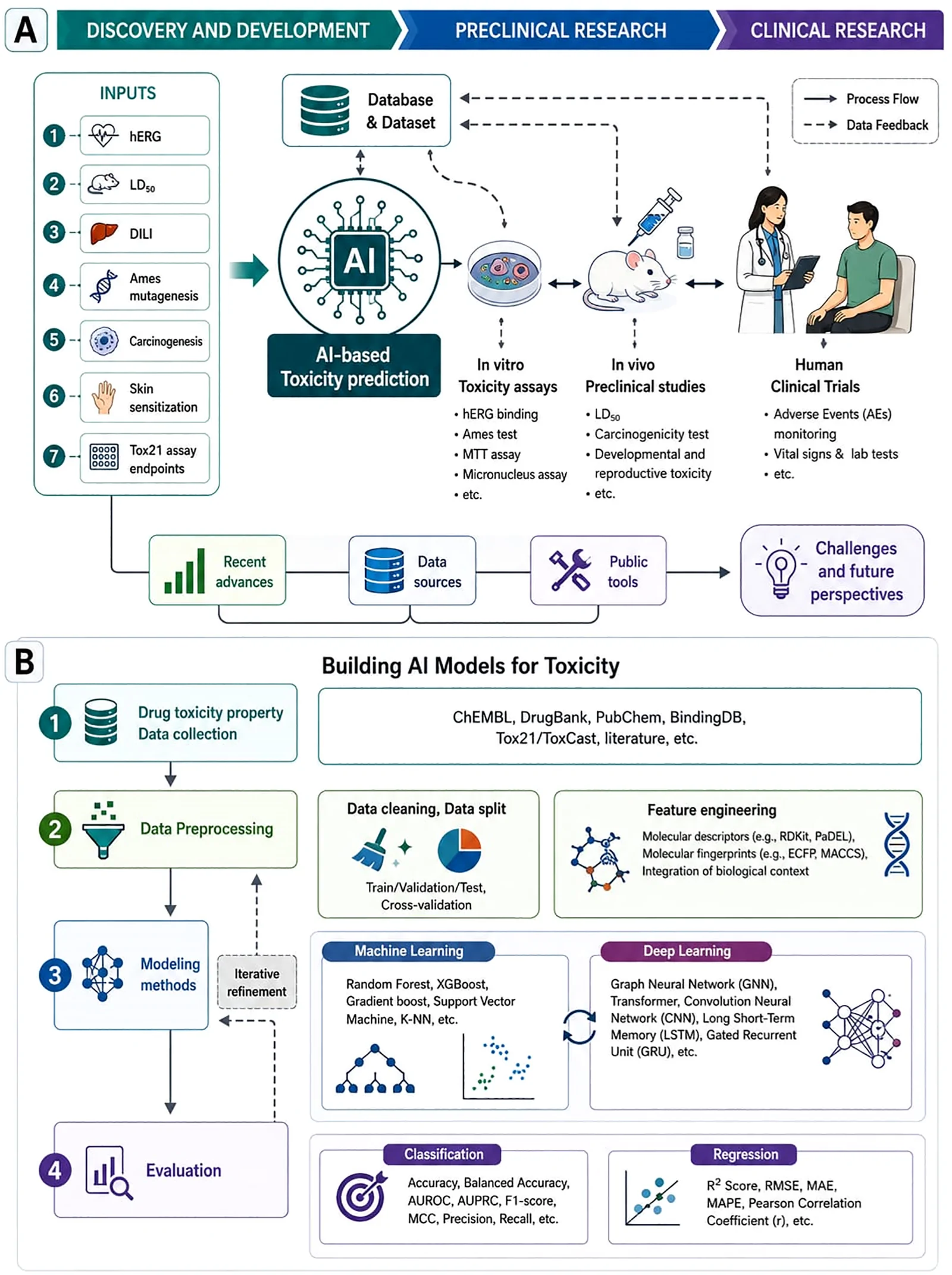
Overview of the AI-based toxicity prediction pipeline. **(A)** Integration of AI-based toxicity prediction. **(B)** Workflow of model development. Source adapted from: Lee et al. (65). Recreated by: Canva, Luna (https://lunaaiart.app), and BioRender (https://www.biorender.com).

### 6.2 Data sources for AI models

AI models are trained on large public databases (ChEMBL) and proprietary *in vitro*, *in vivo*, clinical-trial, and post-marketing surveillance datasets (63, 97). Incorporating AI-based toxicity prediction into virtual screening and then using the experimental results from downstream studies creates a beneficial cycle. This feedback loop includes both prospective and independent validations, which assess model performance with newly generated data. Prospective and independent validations are essential to demonstrate generalizability and meet regulatory expectations (63). This continuous feedback improves model accuracy over time and supports better decisions in early toxicity assessments.

### 6.3 Safety and role in lead optimization

Safety drives attrition in drug development; early ADMET and toxicity prediction reduce clinical failure risk (64, 95). AI-based models have recently become useful for predicting toxicity during early lead optimization. Advances in ADMET datasets, compute, and AI methods (including graph neural networks and message-passing architectures) have improved predictive performance for many endpoints (64, 96). They provide improved predictions for major toxicological endpoints and cover more ground (64, 65, 96).

### 6.4 Available ADMET tools

Several publicly accessible ADMET prediction tools are available, including ADMETLab 3.0, Deep-PK, ProTox 3.0, Helix-ADMET, FP-ADMET, and admetSAR 2.0, each differing in scope, algorithms, and coverage; many provide uncertainty estimates and graph-based or message-passing neural network implementations. ADMETLab 3.0: Multi-endpoint ADMET predictions and profiling (65). Deep-PK uses deep learning to predict multiple endpoints including toxicity and pharmacokinetic properties, and provides explanatory subgraph visualizations (98). ProTox 3.0 provides many endpoint models and incorporates mechanistic insights via adverse outcome pathways. Helix-ADMET employs self-supervised and multitask learning for endpoint extensibility and improved generalizability (99). FP-ADMET uses random forest classifiers trained on many fingerprint types to cover chemical space (Venkatraman, 2021). AdmetSAR 2.0 provides curated endpoints and applies algorithms such as RF, SVM, and k-NN (100). These tools vary by algorithm, endpoints covered, applicability domain, and whether they provide uncertainty estimates or mechanistic explanations.

### 6.5 Utility and limits of general purpose tools

These tools are very useful for ADMET screening because they led to prioritize drug candidates with good safety profiles. The creation of these general-purpose prediction tools has been mostly made possible by improvements in molecular representations that accurately capture compound features and the availability of benchmark datasets with a wide range of ADMET endpoints. Conversely, instruments concentrating on toxicity categories, including hepatotoxicity, cardiotoxicity, nephrotoxicity, neurotoxicity, and genotoxicity/carcinogenicity, frequently necessitate task-specific datasets and customized feature engineering methodologies to improve predictive efficacy.

### 6.6 Explainable AI for toxicity

Explainable AI (XAI) addresses the black-box concern by providing interpretable rationales for toxicity predictions, which are critical for medicinal chemists, toxicologists, and regulators. XAI methods used in toxicity modeling include feature-attribution techniques such as SHAP and Integrated Gradients to highlight molecular substructures or descriptors driving predictions, and subgraph or saliency explanations in graph neural networks that localize toxicophores or reactive motifs (98). Mechanistic linking to adverse outcome pathways (AOPs) enables models to map predicted perturbations to known biological cascades while counterfactual and prototype explanations show minimal structural changes that flip toxicity predictions, thereby aiding design decisions. Incorporating XAI improves trust, supports hypothesis generation such as identifying off-target liabilities and helps prioritize experimental follow-up (67, 68).

### 6.7 Regulatory acceptance and validation

Regulatory acceptance of AI toxicity tools is emerging but requires rigorous evidence. An established evidence hierarchy progress from internal cross-validation to external blinded validation, then to prospective experimental confirmation, and finally to regulatory submission supporting decision-making (63). Regulators expect transparent documentation including training data provenance, preprocessing, applicability domain, performance metrics, uncertainty quantification, and identified failure modes. Currently, regulators accept in silico tools primarily for screening and prioritization rather than as sole evidentiary support for safety decisions; integration with traditional testing remains the prevailing pathway (63, 64). Initiatives including consortia and regulatory pilots such as the OECD framework, OECD AOP framework, and regional regulatory pilots are beginning to evaluate computational models and standardise acceptance criteria. Early engagement with regulators and designing prospective validation studies aligned with regulatory standards will accelerate clinical and regulatory adoption (63, 72).

### 6.8 Future directions and collaboration needs

Future research should focus on integrating various data types, combining chemical structure data with high-resolution biological assay results, multiple omics readouts, and clinical evidence to capture complex toxic mechanisms. This integrated approach can more accurately reflect the complex biology behind toxic responses (64). It is also important to make sure that toxicity annotations are standardize across all databases so that they can work together better and be used repeatedly. Finally, it will be very important to strengthen collaboration between computational scientists, toxicologists, medicinal chemists, clinical pharmacologists, and regulatory stakeholders. This will help create models that are both accurate and easy to understand, which will help make decisions clear across discovery and regulatory content.

## 7 Artificial intelligence in cancer immunotherapy

Cancer immunotherapy harness the immune system to control or eliminate tumors and complements chemotherapy, radiotherapy, and surgery (101, 102). However, these therapies do not work for everyone, and a significant number of patients either do not respond or only show partial responses (20). AI can detect patterns, predict how patients will respond, and aid in creating more personalized and effective treatment plans by analyzing large datasets This combination of immunotherapy and AI has great potential for advancing cancer care, offering the chance for more precise, targeted, and successful interventions to improve patient outcomes and quality of life. AI is increasingly integrated into cancer immunotherapy, providing unmatched opportunities to enhance treatment effectiveness, predict patient responses, and identify new therapeutic targets (70). AI is set to play a vital role in refining these therapies, leading to more personalized and effective patient care. Common AI applications include image segmentation, biomarker discovery (refer **Figure 4**), decision support, dose and plain optimization and quality assurance, (103).

**Figure 4:**
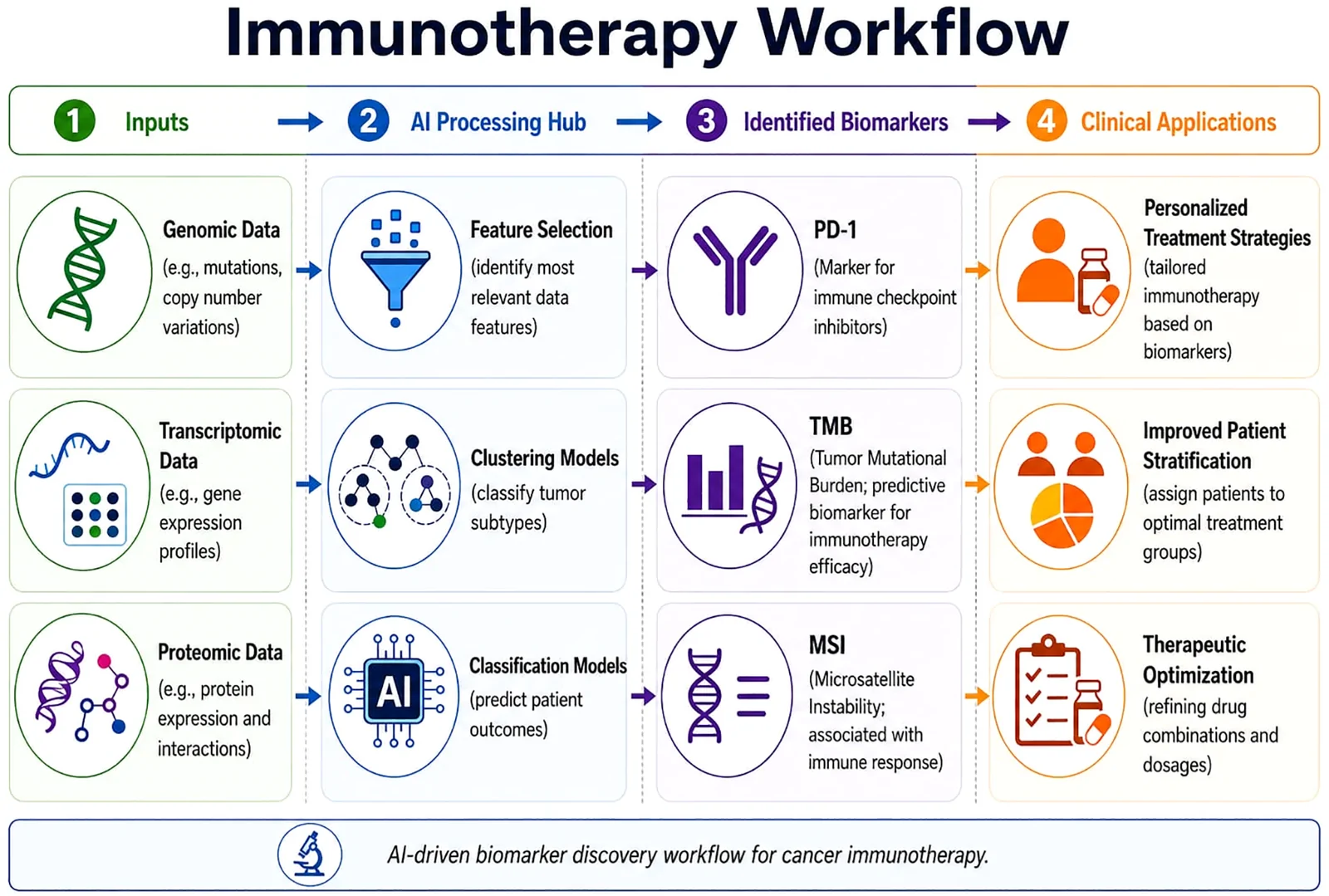
AI-driven biomarker discovery workflow for cancer immunotherapy. Source adapted from: Olawade et al. (70). Recreated by Canva, Luna (https://lunaaiart.app), and BioRender (https://www.biorender.com).

### 7.1 AI-driven biomarker discovery workflow

The workflow begins with multimodal data collection from various sources, including genomic sequences, patient medical records, transcriptomics, proteomics, immune profiling (flow cytometry/CyTOF), ctDNA and imaging data. These datasets are analyzed, processed, and integrated into AI and machine learning models. The models are trained to find patterns that assist doctors in deciding on immunotherapy treatments. This personalized approach allows for tailored treatment plans for each patient. The system includes a feedback loop that tracks patient outcomes and returns that information to the AI, which helps continually refine healthcare delivery. This iterative process boosts the accuracy of cancer immunotherapy, leading to more precise and effective treatment strategies (70). **Table 6** presents a comprehensive overview of the diverse applications of AI in advancing cancer immunotherapy. It highlights how AI supports the development of more effective and personalized treatment strategies by outlining key methodologies, specific examples, and their corresponding impacts. Overall, the table serves as a valuable resource for understanding the breadth and potential of AI in this domain.

**Table 6:** Comprehensive overview of AI applications in cancer immunotherapy.

S.No	Application	Description	Methodology	Examples	Impact on treatment
1.	Biomarker Identification	AI analyzes complex biological data to discover biomarkers that predict immunotherapy response	Supervised ML algorithms (e.g., Random Forest, Support Vector Machines) and DL algorithms (e.g., Convolutional Neural Networks, Recurrent Neural Networks) applied to multi-omics data (genomic, transcriptomic, proteomic)	Identification of TMB, MSI, PD-L1 expression levels, and gene signatures predictive of ICI response	Enables personalized treatment by stratifying patients based on likely treatment response, improving efficacy, and minimizing adverse effects
2.	Optimization of Combination Therapies	AI predicts the most effective combinations of immunotherapy with other treatments (e.g., chemotherapy, radiation)	Reinforcement learning models and Bayesian networks applied to clinical trial data, real-world evidence, and patient-specific data	Successful combinations of ICIs with chemotherapy or radiation therapy in lung cancer and melanoma	Reduces reliance on trial and error, accelerates the identification of optimal treatment regimens, and enhances overall therapeutic outcomes
3.	Predicting Patient Responses	AI models forecast which patients will benefit from specific immunotherapy treatments and who might experience severe side effects	Gradient Boosting Machines, Logistic Regression, and DL models (e.g., Multi-Layer Perceptrons) using patient data (e.g., genomic profiles, immune signatures, imaging data)	ML predictions for PD-1/PD-L1 inhibitor responses in melanoma and non-small cell lung cancer	Guides clinical decision-making, allowing for more precise, personalised treatment and avoiding unnecessary side effects
4.	Discovering Novel Therapeutic Targets	AI uncovers new targets for immunotherapy by analyzing vast biological datasets	Unsupervised ML models (e.g., clustering and principal component analysis) and Generative Adversarial Networks (GANs) applied to genetic, epigenetic, and transcriptomic data	Identification of neoantigens and novel ICIs for developing personalized vaccines and new therapeutic agents	Expands the range of therapeutic options, leading to the development of new immunotherapy agents tailored to specific tumor types
5.	Enhancing Drug Discovery	AI accelerates the discovery and development of new immunotherapeutic agents	Virtual screening, DL molecular modeling (e.g., Graph Neural Networks), and simulation of drug interactions	AI-driven discovery of novel checkpoint inhibitors and monoclonal antibodies for cancer treatment	Shortens the drug development timeline, reducing costs and bringing effective treatments to market more quickly
6.	Monitoring Treatment Response	AI facilitates real-time monitoring of patient responses during immunotherapy	Time-series analysis models and DL algorithms (e.g., Long Short-Term Memory networks) applied to data from wearables, imaging, and EHR	AI-integrated wearable devices monitor physiological changes and detect early signs of adverse reactions	Enables timely interventions, optimizing treatment outcomes and improving patient safety during therapy
7.	Improving Patient Selection for Clinical Trials	AI identifies suitable candidates for clinical trials based on predictive models	Natural Language Processing (NLP), supervised ML models (e.g., Decision Trees), and DL models (e.g., Transformers) applied to EHR, genetic profiles, and clinical history	Enhanced recruitment for immunotherapy trials, particularly for rare cancer types or specific genetic subtypes	Increases the efficiency and success rate of clinical trials, ensuring that trials are populated with the most suitable candidates
8.	Adaptive Treatment Strategies	AI supports dynamic adjustment of treatment plans based on ongoing patient data	Adaptive reinforcement learning and real-time DL models integrating data from multiple sources for adjusting treatment parameters	AI-driven adaptive dosing and sequencing strategies in immunotherapy to enhance effectiveness and reduce toxicity	Personalizes treatment regimens, improving outcomes by adapting to individual patient responses over time

Source: Adapted and Reproduced from Olawade et al. (70) "Integrating AI into cancer immunotherapy - A narrative review of current applications and future directions".

### 7.2 Artificial intelligence in cancer immunotherapy: key advances

#### 7.2.1 Neoantigen prediction

Neoantigens are tumor-specific peptides derived from somatic mutations are central to personalized cancer vaccines and adoptive T-cell therapies. AI improves neoantigen pipelines by:

- Predicting peptide–MHC binding using deep learning models trained on mass-spectrometry eluted ligands and binding assays, improving MHC-I/II accuracy and reducing candidate lists for experimental validation.
- Modeling antigen processing and presentation (proteasomal cleavage, TAP transport) and estimating T-cell receptor (TCR) recognition probabilities.
- Prioritizing neoantigens by integrating predicted presentation, clonality, expression level, and similarity to self to increase the likelihood of clinically relevant targets (70, 103, 104).
- Integration with RNA expression and clonality metrics further refines selection for clinical use (70).

#### 7.2.2 Tumor microenvironment modeling and immune landscape

AI integrates bulk and single-cell omics, spatial transcriptomics, and imaging to characterize the TME immune composition, activation states, and spatial organization.

The Applications include:

- Deconvolution of bulk RNA to estimate immune infiltrates and cell subtypes predictive of immunotherapy benefit.
- Single-cell clustering and trajectory analysis to identify exhausted T-cell states, myeloid suppressor populations, and stromal interactions linked to resistance.
- Spatial models quantifying neighborhoods (e.g., CD8+ T cell proximity to tumor cells) and tertiary lymphoid structures associated with response.
- These insights inform combination strategies (e.g., checkpoint blockade + myeloid targeting) and patient selection (13, 42, 70).

#### 7.2.3 Immune checkpoint prediction and response modeling

AI predicts how well patients will respond to immune-checkpoint inhibitors (ICIs) by combining multiple types of information: the number of mutations in the tumor (tumor mutational burden, or TMB), the level of PD-L1 protein on cells, gene-expression patterns, the amount of neoantigens (new tumor-specific proteins), features of the tumor microenvironment (such as immune cell presence), and imaging data from scans (radiomics). Models that use many of these data types together (multimodal models) capture more complete biological information and work better than models that rely on just one type of data. These AI models can also estimate the risk of immune-related adverse events (irAEs), unwanted side effects caused by overactive immune responses, by analyzing clinical history, genetic data, and information about previous treatments (70, 103–105).

#### 7.2.4 Spatial transcriptomics and multimodal spatial analysis

Spatial transcriptomics, combined with histology (tissue imaging), maps where genes are expressed in their tissue context. AI pipelines align spatial gene expression data with histology images using image registration and multimodal deep learning. These tools identify spatial patterns of cell–cell interactions that predict whether a patient will respond to or resist therapy. They also discover distinct micro-niches in tumors, such as immune-excluded, immune-inflamed, or immune-desert regions, that help guide treatment choices. Combining spatial data with single-cell analyses refines biological hypotheses and improves biomarker selection for clinical trials (13, 42, 70).

#### 7.2.5 AI in vaccine design and immunotherapy scheduling

AI optimizes vaccine design by prioritizing neoantigens (tumor-specific targets), designing peptide sequences for better stability and immune presentation, selecting appropriate adjuvants, and determining optimal vaccination schedules to maximize immune response. Simulation models and digital twins allow researchers to test different scheduling and combination treatment strategies in silico (on computers) before moving to clinical trials, helping to identify the most promising approaches (70, 103).

## 8 AI in CAR-T and biomarker discovery

The efficacy of immunotherapy is variable between patients, which is due to the complex and diverse nature of the tumor (66, 106). Also, we see that AI has played a very key role in the field of Chimeric Antigen Receptor T cell therapy in terms of improving the design and optimization of CAR (14, 73). By use of advanced algorithms, AI enables us to identify what we term optimal target antigens and to predict CAR-T cell performance, which in turn improves safety and efficacy. Also, we see that AI is used to great effect in the design of CAR constructs, which in turn reduces time and costs associated with the traditional trial and error methods Also, out of AI has come the identification of biomarkers which play a key role in the response to immunotherapy, which in turn is very important for the personalization of treatment and improving results.AI models can integrate these biomarkers into predictive algorithms, helping clinicians to stratify patients and select those who are most likely to benefit from immunotherapy (103, 104).

Artificial intelligence accelerates chimeric antigen receptor T-cell (CAR-T) development across multiple design steps. Machine learning and network-based methods mine tumor expression profiles, normal tissue atlases, and single-cell datasets to identify tumor-restricted target antigens, thereby minimizing on-target off-tumor toxicity. Deep learning models predict the binding affinity and specificity of scFv sequences, enabling in silico optimization of CAR binding domains. AI further explores linker length, co-stimulatory domain choices, and expression scaffolds to improve persistence, proliferation, and safety by reducing tonic signaling. In silico assessment of cross-reactivity and off-target binding using proteome-wide screening models reduces the risk of unforeseen toxicities. Collectively, these tools shorten design cycles and focus experimental testing on higher-probability constructs (14, 73). AI integrates tumour antigen density, HLA context, microenvironmental suppression, and patient immune fitness to predict CAR-T expansion, persistence, and the likelihood of durable response. Models can flag patients at higher risk of severe cytokine release syndrome or neurotoxicity, guiding prophylaxis and monitoring strategies.

Beyond CAR-T, AI supports TCR-T therapy by predicting peptide–MHC presentation and TCR–peptide recognition, enabling selection of naturally presented neoepitopes and TCRs with favorable specificity. Combining repertoire sequencing with machine learning helps identify tumor-reactive clonotypes for expansion.AI identifies composite biomarkers that combine genomic alterations, tumor microenvironment features, spatial patterns, and peripheral immune signatures to stratify patients for specific immunotherapies or combinations. Predictive models are increasingly integrated into tumor-board workflows to support therapy selection and trial matching (103, 104).

Spatial transcriptomics and single-cell profiling reveal barriers to CAR-T infiltration, including stromal barriers and suppressive myeloid niches. AI models predict which tumors are amenable to CAR-T infiltration and suggest engineering strategies, such as chemokine receptor modification, to improve trafficking. While many AI immunotherapy models show promising retrospective performance, prospective validation, controlled trials, and external multicenter testing are required to establish clinical utility. Embedding AI predictions into adaptive trials and collecting outcome feedback accelerate model refinement and clinical acceptance.

AI enhances cancer immunotherapy by improving target discovery, neoantigen prioritization, tumor microenvironment modeling, spatial and single-cell integration, CAR-T design, and biomarker development. Real clinical benefit requires prospective validation, integration into care pathways, and careful attention to interpretability, safety, and ethical considerations.

## 9 Challenges and future directions

### 9.1 Key barriers to clinical translation

However, high-quality, well-annotated data sets are fragmented, skewed towards specific populations and tumor types, and heterogeneous in terms of format and pre-processing, which limits the generalizability of models to new settings (64, 107). Black-box models are hard for clinicians to trust and for regulators to review, so explainable AI (XAI) is needed to show mechanistic plausibility and actionable reasons for predictions (67, 68). Most models are validated retrospectively or on internal cohorts only; prospective multicenter validation is a rarity. Once in production, models can suffer from model drift over time due to changes in assays, patient populations or clinical practice requiring ongoing monitoring, recalibration and governance. Regulatory acceptance is still in its infancy, and only a handful of AI tools have FDA or CE clearance, and regulators look for clear reporting on data provenance, applicability domain, uncertainty quantification and prospective evidence in line with standards such as ISO 13485 and IEC 62304 (63). Large-scale training and external validation are hampered by privacy laws and institutional policies that restrict data pooling. Finally, algorithmic bias could exacerbate health disparities if training datasets do not adequately represent some demographic or socioeconomic groups (108, Kleinberg, 2018).

### 9.2 Technical and methodological directions

Federated learning enables the training of models across multiple sites without the need for sharing raw patient data, allowing for diverse training cohorts while respecting privacy. Successful adoption requires harmonized feature sets, robust aggregation, and safeguards against client-side bias or model poisoning. Generative models can help augment scarce datasets (e.g. chemical libraries, rare tumor subtypes, single-cell profiles) to mitigate sampling bias and support stress-testing. However, synthetic data requires validation to preserve real biological correlations and avoid amplification of artifacts (109). Large pretrained biomedical foundation models and multimodal transformers improve transferability across tasks and reduce the need for large, labeled datasets, but require careful fine-tuning, calibration and external benchmarking before clinical use. Pipelines should include XAI techniques (feature attributions, counterfactuals, prototype explanations) and robust uncertainty estimates (calibration, Bayesian) so that clinicians and regulators can interpret and weigh model outputs. Continuously monitor: Track systems for performance drift, use versioning and reproducible pipelines, establish pre-defined retraining policies, and establish multidisciplinary governance boards (clinicians, data scientists, ethicists) for oversight.

### 9.3 Regulatory science and AI governance

The recommended validation framework is tiered, including internal validation, external retrospective validation, prospective preclinical/clinical validation, and regulatory submission with post-market surveillance. Regulators are increasingly asking for documentation of training data provenance, bias assessment, applicability domain, and post-deployment monitoring plans. Engagement with regulators early on and involvement in pilot programs and consortia (eg, OECD, regional regulatory initiatives) standardize benchmarks and acceptance criteria. Development should be in accordance with medical device and software standards (ISO 13485, IEC 62304) for quality management and software lifecycle. Governance frameworks should be transparent, covering data stewardship, patient consent, fairness auditing, explainability thresholds, and incident response. Public registries of deployed clinical AI models (with versioning and intended use) improve accountability.

### 9.4 Implementation and workforce considerations

AI must be integrated into clinical workflows with usability studies, human–AI interaction design, and explainability tailored to end users (oncologists, pathologists, trialists). AI outputs should be embedded into tumor-board workflows with clear actionability statements. Clinicians and trial staff need training in AI literacy, uncertainty interpretation, and data stewardship, and cross-disciplinary teams should be promoted for development, validation, and governance. Sustained partnerships among computational scientists, bench researchers, clinicians, regulators, and patient representatives are essential to designing clinically relevant studies and shared validation efforts.

### 9.5 Ethical and social problems

Diverse populations must be represented in datasets, consent models must enable responsible reuse, and communication with patients about AI-assisted decisions must be transparent. “Rapid development needs to be balanced with rigorous validation so that premature clinical translation and patient harm do not occur.

### 9.6 Practical recommendations

- Follow a hierarchy of validation: internal, external, prospective multicenter, regulatory submission with post-market monitoring.
- Use federated learning and synthetic data judiciously to expand training sets without sacrificing data fidelity.
- Demand XAI and uncertainty reporting for any model at clinical grade
- Engage regulators early and adopt device/software quality management standards.Continuous monitoring and governance to detect model drift and maintain safety.

### 9.7 Paradigm shift in oncology drug management

AI is driving a profound paradigm shift in oncology drug management. From drug discovery and development to clinical application and post-marketing surveillance, AI offers unprecedented gains in efficiency and decision-making support (110). In this scenario, one of the most promising expectancies for AI is the possibility of integrating different and composite data derived from multidimensional approaches for patients with cancer (16).

### 9.8 Pitfalls and overhype

Despite all the promises of AI technology, there are formidable obstacles and pitfalls. The state of AI hype has far exceeded the state of AI science, especially when it pertains to validation and readiness for implementation in patient care. A recent example is IBM Watson Health’s cancer AI algorithm (known as Watson for Oncology) used by hundreds of hospitals around the world for recommending treatments for patients with cancer, the algorithm was based on a small number of synthetic, nonreal cases with very limited input (real data) of oncologists Many of the actual output recommendations for treatment were shown to be erroneous, such as suggesting the use of bevacizumab in a patient with severe bleeding, which represents an explicit contraindication and ‘black box’ warning for the drug.

### 9.9 Data availability and quality challenges

Despite the potential benefits of AI in drug discovery, there are several challenges and limitations that must be considered. One of the key challenges is the availability of suitable (64). AI-based methods usually need a lot of data for training purposes (111). Often, the available data is limited, low quality, or inconsistent, affecting the accuracy and reliability of results (Gómez-Bombarelli et al., 2018). Ethical considerations also present challenges as AI approaches may raise concerns about fairness and bias (108, Kleinberg, 2018).

### 9.10 Mitigation strategies

Several strategies can overcome the obstacles faced by AI in chemical medicine. One approach involves data augmentation (109), which generates synthetic data to supplement existing datasets. This increases the volume and diversity of data available for training machine learning algorithms, improving accuracy and reliability. Another strategy is to use explainable AI methods, which aim to provide clear explanations for the predictions made by algorithms (67). This can help address bias concerns and give a better understanding of the mechanisms and assumptions behind predictions (67; Kleinberg, 2018).

### 9.11 Complementary role of human expertise

Current AI methods are not a replacement for traditional experimental methods and cannot replace the expertise of human researchers (112, 113). AI can only make predictions based on available data, which human researchers must then validate (68). However, combining AI with traditional methods can enhance the drug discovery process. By leveraging AI’s predictive power together with human expertise (114), we can improve and speed up the development of new medications (115). The initial feasibility of computationally guided therapeutic discovery exists, yet moving from algorithm-based design to delivering clinical benefits is hindered by barriers like generalizability, interpretability, equity, regulatory harmonization, and the need for targeted mitigation strategies (72).

## 10 Conclusion

AI has demonstrable utility across anticancer drug discovery and precision oncology including target identification, molecular design, ADMET prediction, biomarker discovery, and clinical trial optimization but its clinical impact depends on rigorous validation and integration with experimental workflows. Major translational gaps include fragmented data, limited external and prospective validation, interpretability shortfalls, regulatory uncertainty, and potential biases. Well-validated AI tools can accelerate candidate selection, personalize therapy, improve trial efficiency, and enable real-time safety monitoring, though clinicians must interpret AI outputs with awareness of their limitations and uncertainty. Progress requires federated, privacy-preserving data sharing; foundation and multimodal models with external benchmarking; explainable and uncertainty-aware systems; and regulatory science to define evidence pathways. With coordinated technical, clinical, and regulatory efforts, AI can safely and sustainably transform oncology care over the next decade.

## References

[1] Cao W Qin K Li F Chen W . Comparative study of cancer profiles between 2020 and 2022 using global cancer statistics (GLOBOCAN). J Natl Cancer Center. (2024) 4:128–34. doi: 10.1016/j.jncc.2023.12.001 39282581 PMC11390618

[2] Khan SU Fatima K Aisha S Malik F . Unveiling the mechanisms and challenges of cancer drug resistance. Cell Commun Signaling. (2024) 22:109. doi: 10.1186/s12964-023-01302-1 38347575 PMC10860306

[3] Iwaloye O Ottu PO Olawale F Babalola OO Elekofehinti OO Kikiowo B . Computer-aided drug design in anti-cancer drug discovery: What have we learnt and what is the way forward? Inf Med Unlocked. (2023) 41:101332. doi: 10.1016/j.imu.2023.10133242574925

[4] Schuhmacher A Gassmann O Hinder M . A review of the pharmaceutical R&D efficiency: Costs, timelines, and probabilities. In: Schuhmacher A Gassmann O Hinder M , editors. Value Creation in the Pharmaceutical Industry: The Critical Path to Innovation. Weinheim: Wiley-VCH Verlag GmbH & Co. KGaA (2016). p. 60–79. doi: 10.1002/9783527693405.ch4

[5] Swanson K Walther P Leitz J Mukherjee S Wu JC Shivnaraine RV . ADMET-AI: A machine learning ADMET platform for evaluation of large-scale chemical libraries. Bioinformatics. (2024) 40:btae416. doi: 10.1093/bioinformatics/btae416 38913862 PMC11226862

[6] Albani FG Alghamdi SS Almutairi MM Alqahtani T . Artificial intelligence-driven innovations in oncology drug discovery: Transforming traditional pipelines and enhancing drug design. Drug Des Dev Ther. (2025) 19:5685–707. doi: 10.2147/DDDT.S509769 40626099 PMC12232943

[7] Hu Y Stumpfe D Bajorath J . Lessons learned from molecular scaffold analysis. J Chem Inf Model. (2011) 51:1742–50. doi: 10.1021/ci200179y 21755989

[8] Bender A Cortés-Ciriano I . Artificial intelligence in drug discovery: What is realistic, what are illusions? Part 2: A discussion of chemical and biological data. Drug Discov Today. (2021) 26:1040–52. doi: 10.1016/j.drudis.2020.11.037 33508423 PMC8132984

[9] Luchini C Pea A Scarpa A . Artificial intelligence in oncology: Current applications and future perspectives. Br J Cancer. (2022) 126:4–9. doi: 10.1038/s41416-021-01633-1 34837074 PMC8727615

[10] Pathak A Theagarajan R Rizqi MM Nugraha AS Boruah T Kumar H . AI-enabled drug and molecular discovery: Computational methods, platforms, and translational horizons. Discover Molecules. (2025) 2:32. doi: 10.1007/s44345-025-00037-530311153

[11] Dlamini Z Francies FZ Hull R Marima R . Artificial intelligence (AI) and big data in cancer and precision oncology. Comput Struct Biotechnol J. (2020) 18:2300–11. doi: 10.1016/j.csbj.2020.08.019 PMC7490765 32994889

[12] Ali H . Artificial intelligence in multi-omics data integration: Advancing precision medicine, biomarker discovery and genomic-driven disease interventions. Int J Sci Res Archival. (2023) 8:1012–30. doi: 10.30574/ijsra.2023.8.1.0189

[13] Bera K Schalper KA Rimm DL Velcheti V Madabhushi A . Artificial intelligence in digital pathology—New tools for diagnosis and precision oncology. Nat Rev Clin Oncol. (2019) 16:703–15. doi: 10.1038/s41571-019-0252-y 31399699 PMC6880861

[14] Bujak JK Lee HJ Daghestani HN colleagues . Artificial intelligence in CAR-T cell engineering and precision immunotherapy. Front Immunol. (2023) 14:1206458. doi: 10.3389/fimmu.2023.1206458

[15] Cardoso F Piccart-Gebhart M Van't Veer L Rutgers E TRANSBIG Consortium . The MINDACT trial: the first prospective clinical validation of a genomic tool. Mol Oncol. (2007) 1:246–51. doi: 10.1016/j.molonc.2007.10.004 19383299 PMC5543876

[16] Corti C Cobanaj M Dee EC Criscitiello C Tolaney SM Celi LA . Artificial intelligence in cancer research and precision medicine: Applications, limitations and priorities to drive transformation in the delivery of equitable and unbiased care. Cancer Treat Rev. (2023) 112:102498. doi: 10.1016/j.ctrv.2022.102498 36527795

[17] Desai D Kantliwala SV Vybhavi J Ravi R Patel H Patel J . Review of alphaFold 3: transformative advances in drug design and therapeutics. Cureus. (2024) 16:e63646. doi: 10.7759/cureus.63646 39092344 PMC11292590

[18] Ding T Wagner SJ Song AH Chen RJ Lu MY Zhang Andrew . A multimodal whole-slide foundation model for pathology. Nat Med. (2025) 31:3749–61. doi: 10.1038/s41591-025-03982-3 41193692 PMC12618242

[19] Duffus JH . Toxicology and risk assessment. Pure Appl Chem. (1993) 65:2003–122. doi: 10.1351/pac199365092003

[20] Emens LA Butterfield LH Hodi FS Marincola FM Kaufman HL Cancer Immunotherapy Trials Network . Cancer immunotherapy trials: Leading a paradigm shift in drug development. J Immunother Cancer. (2017) 5:42. doi: 10.1186/s40425-016-0146-9 27437105 PMC4949883

[21] Ferber D El Nahhas OSM Wölflein G Wiest IC Clusmann J Leßmann Marie-Elisabeth . Development and validation of an autonomous artificial intelligence agent for clinical decision-making in oncology. Nat Cancer. (2025) 6:1337–49. doi: 10.1038/s43018-025-00991-6 40481323 PMC12380607

[22] Francies FZ Wainstein T De Leeneer K Cairns A Murdoch M Nietz S . BRCA1, BRCA2 and PALB2 mutations and CHEK2 c.1100delC in different South African ethnic groups diagnosed with premenopausal and/or triple-negative breast cancer. BMC Cancer. (2015) 15:912. doi: 10.1186/s12885-015-1913-6 26577449 PMC4647511

[23] Ali S Tian X Chen H Zhou J . A new era of artificial intelligence (AI): Transforming drug discovery and development. J Med Chem. (2025) 68(22):23643–52. doi: 10.1021/acs.jmedchem.5c03159 PMC12908618 41306069

[24] Nicolaou KC . Advancing the drug discovery and development process. Angewandte Chemie International Edition. (2014) 53(35):9128–40. doi: 10.1002/anie.201404761 25045053

[25] Sali R Aschenaki Y Leveillee R Baba F Tessema L Dixon C . Foundation models in computational pathology: Methods, applications and clinical implications. BMJ Oncol. (2026) 5:e001102. doi: 10.1136/bmjonc-2026-001102 42117016 PMC13157769

[26] Pei J Zhang J Cong Q . Computational analysis of protein-protein interactions of cancer drivers in renal cell carcinoma. FEBS Open Bio. (2024) 14:112–26. doi: 10.1002/2211-5463.13732 37964489 PMC10761929

[27] Li MQC Wang S Lin SR Ting LEN Wan ZH Xie G . Advantages and limitations of AlphaFold in structural biology: Insights from recent studies. Protein J. (2026) 45:22–38. doi: 10.1007/s10930-025-10310-8 41326937 PMC12956938

[28] Niazi SK . Artificial intelligence in small-molecule drug discovery: A critical review of methods, applications, and real-world outcomes. Pharmaceuticals. (2025) 18:1271. doi: 10.3390/ph18091271 41011141 PMC12472608

[29] Pushpakom S Iorio F Eyers P Battaglinzo S Dunning M Delpin M . Drug repurposing: Progress, challenges and recommendations. Nat Rev Drug Discov. (2019) 18:41–57. doi: 10.1038/nrd.2018.168 30310233

[30] Fu Z Yang Y Chung MK Cheng F . Artificial intelligence to guide repurposing of drugs. Annu Rev Med. (2026) 77:381–98. doi: 10.1146/annurev-med-050224-122802 41592930 PMC12854511

[31] Zhou Y Hou Y Shen J Huang Y Martin W Cheng F . Network-based drug repurposing for novel coronavirus 2019-nCoV/SARS-CoV-2. Cell Discov. (2020) 6:14. doi: 10.1038/s41421-020-0153-3 32194980 PMC7073332

[32] Keramida P Syrigos NK Kouvela M Poulakou G Charpidou A Fiste O . AI-driven drug repurposing: applications and challenges. Medicines. (2025) 12:28. doi: 10.3390/medicines12040028 41283338 PMC12641654

[33] Ashley EA . Towards precision medicine. Nat Rev Genet. (2016) 17:507–22. doi: 10.1038/nrg.2016.86 27528417

[34] Bhalla S Laganà A . Artificial intelligence for precision oncology. In Laganà A. (Ed.), Computational methods for precision oncology (2022) (pp. 249–68). Springer. doi: 10.1007/978-3-030-91836-1_14 35230693

[35] Niazi MKK Parwani AV Gurcan MN . Digital pathology and artificial intelligence. Lancet Oncol. (2019) 20:e253–61. doi: 10.1016/S1470-2045(19)30154-8 31044723 PMC8711251

[36] Ramwala OA Lowry KP Cross NM Hsu W Austin CC Mooney SD . Establishing a validation infrastructure for imaging-based artificial intelligence algorithms before clinical implementation. Journal of the American College of Radiology, (2024) 21(10):1569–74. doi: 10.1016/j.jacr.2024.04.027 PMC11486600 38789066

[37] Suleman MU Mursaleen M Khalil U Saboor A Bilal M Khan SA . Assessing the generalizability of artificial intelligence in radiology: A systematic review of performance across different clinical settings. Ann Med Surg. (2025) 87:8803–11. doi: 10.1097/MS9.0000000000004166 41377327 PMC12689012

[38] Ahmad Z Rahim S Zubair M Abdul-Ghafar J . The new era of precision oncology: Practicing pathology in low-resource countries and settings. International Journal of General Medicine, (2026) 19:593156. doi: 10.2147/IJGM.S593156 PMC13033194 41913905

[39] Neri E Aghakhanyan G Zerunian M Gandolfo N Grassi R Miele V . Explainable AI in radiology: A white paper of the Italian Society of Medical and Interventional Radiology. Radiologia Med. (2023) 128:755–64. doi: 10.1007/s11547-023-01634-5 37155000 PMC10264482

[40] He W Huang W Zhang L Wu X Zhang S Zhang B . Radiogenomics: bridging the gap between imaging and genomics for precision oncology. MedComm. (2024) 5:e722. doi: 10.1002/mco2.722 39252824 PMC11381657

[41] Trivizakis E Papadakis GZ Souglakos I Papanikolaou N Koumakis L Spandidos DA . Artificial intelligence radiogenomics for advancing precision and effectiveness in oncologic care (Review). Int J Oncol. (2020) 57:43–53. doi: 10.3892/ijo.2020.5063 32467997 PMC7252460

[42] Hegde N Hipp JD Liu Y Emmert-Buck M Reif E Smilkov D . Similar image search for histopathology: SMILY. NPJ Digital Med. (2019) 2:56. doi: 10.1038/s41746-019-0131-z 31304402 PMC6588631

[43] Griffin J Treanor D . Digital pathology in clinical use: Where are we now and what is holding us back? Histopathology. (2017) 70:134–45. doi: 10.1111/his.12993 27960232

[44] Park KC Yoo W . Translating multimodal foundation models into oncology: Toward a future where AI directs diagnosis and therapy. Genes & Diseases, (2026) 13(4):101958. doi: 10.1016/j.gendis.2025.101958 PMC12989828 41847347

[45] Ramwala OA Lowry KP Cross NM Hsu W Austin CC Mooney SD . Establishing a validation infrastructure for imaging-based artificial intelligence algorithms before clinical implementation. J Am Coll Radiol. (2024) 21:1569–74. doi: 10.1016/j.jacr.2024.04.027 38789066 PMC11486600

[46] Somashekhar SP Sepúlveda M-J Puglielli S Norden AD Shortliffe EH Kumar CR . Watson for Oncology and breast cancer treatment recommendations: Agreement with an expert multidisciplinary tumour board. Ann Oncol. (2018) 29:418–23. doi: 10.1093/annonc/mdx781 29324970

[47] Somashekhar SP Sepúlveda M-J Shortliffe EH Rohit Kumar C Rauthan A Patil P . A prospective blinded study of 1,000 cases analyzing the role of artificial intelligence: Watson for Oncology and change in decision making of a multidisciplinary tumor board from a tertiary care cancer center. Journal of Clinical Oncology, (2019) 37(15_suppl):6533. doi: 10.1200/JCO.2019.37.15_suppl.6533

[48] Tamborero D Dienstmann R Rachid MH Boekel J Baird R Braña I . Support systems to guide clinical decision-making in precision oncology: The Cancer Core Europe Molecular Tumor Board Portal. Nat Med. (2020) 26:992–4. doi: 10.1038/s41591-020-0969-2 32632195

[49] You H-S Gao C-X Wang H-B Luo S-S Chen S-Y Dong Y-L . Concordance of treatment recommendations for metastatic non-small-cell lung cancer between Watson for Oncology system and medical team. Cancer Manage Res. (2020) 12:1947–58. doi: 10.2147/CMAR.S244932 32214852 PMC7083631

[50] Somashekhar SP Kumar SPR Kumar A Patil P Rauthan A . Validation study to assess performance of IBM cognitive computing system Watson for Oncology with Manipal multidisciplinary tumour board for 1,000 consecutive cases: An Indian experience. Annals of Oncology, (2016) 27(Suppl. 9):mdw601.002. doi: 10.1093/annonc/mdw601.002

[51] Zhou J Zeng Z Li L . A meta-analysis of Watson for Oncology in clinical application. Scientific Reports, (2021) 11:5792. doi: 10.1038/s41598-021-84973-5 PMC7952578 33707577

[52] Kamli H Khan NU . Emerging role of ctDNA fragmentomics and epigenetic signatures in the early detection, minimal residual disease assessment, and precision monitoring of renal cell carcinoma. J Cell Mol Med. (2026) 30:e71019. doi: 10.1111/jcmm.71019 41614220 PMC12856527

[53] Liu T Fang Z-Y Zhang Z Yu Y Li M Yin M-Z . A comprehensive overview of graph neural network-based approaches to clustering for spatial transcriptomics. Computational and Structural Biotechnology Journal, (2024) 23:106–28. doi: 10.1016/j.csbj.2023.11.055 PMC10714345 38089467

[54] Gogoshin G Rodin AS . Graph neural networks in cancer and oncology research: Emerging and future trends. Cancers (Basel). (2023) 15:5858. doi: 10.3390/cancers15245858 38136405 PMC10742144

[55] Gao C Yang C Zhang L . A graph neural network-based spatial multi-omics data integration method for deciphering spatial domains. PLoS Comput Biol. (2025) 21:e1013546. doi: 10.1371/journal.pcbi.1013546 41026788 PMC12503342

[56] Seeger N Gutknecht S Zschokke I Fleischmann I Roth N Metzger J . Predictive noninvasive single-nucleotide variation–based biomarker signature for resectable pancreatic cancer: Protocol for a prospective validation study. JMIR Res Protoc. (2024) 13:e54042. doi: 10.2196/54042 38635586 PMC11130767

[57] Barata PC Mendiratta P Heald B Klek S Grivas P Sohal DPS . Targeted next-generation sequencing in men with metastatic prostate cancer: A pilot study. Targeted Oncol. (2018) 13:495–500. doi: 10.1007/s11523-018-0576-z 29974386

[58] Zarkavelis G Kotoula V Kolliou G-A Papadopoulou K Tikas I Karavasilis V . Genetic mapping of pancreatic cancer by targeted next-generation sequencing in a cohort of patients managed with nab-paclitaxel-based chemotherapy or agents targeting the EGFR axis: A retrospective analysis of the Hellenic Cooperative Oncology Group (HeCOG). ESMO Open. (2019) 4:e000525. doi: 10.1136/esmoopen-2019-000525 31673425 PMC6802956

[59] D’Haene N Fontanges Q De Nève N Blanchard O Melendez B Delos M . Clinical application of targeted next-generation sequencing for colorectal cancer patients: A multicentric Belgian experience. Oncotarget. (2018) 9:20761–73. doi: 10.18632/oncotarget.25099 29755687 PMC5945518

[60] Jensen KH Izarzugaza JMG Juncker AS Hansen RB Hansen TF Timshel P . Analysis of a gene panel for targeted sequencing of colorectal cancer samples. Oncotarget. (2018) 9:9043–57. doi: 10.18632/oncotarget.24138 29507673 PMC5823670

[61] Bewicke-Copley F Kumar EA Palladino G Korfi K Wang J . Applications and analysis of targeted genomic sequencing in cancer studies. Comput Struct Biotechnol J. (2019) 17:1348–59. doi: 10.1016/j.csbj.2019.10.004 31762958 PMC6861594

[62] Kong Q Ban Y . AI-driven radiogenomics in gynecologic oncology: From radiological digital biopsy to a new paradigm in precision therapy. Frontiers in Oncology, (2026) 16:1745519. doi: 10.3389/fonc.2026.1745519 PMC12913118 41717418

[63] Pognan F Beilmann M Boonen HCM Czich A Dear G Hewitt P . The evolving role of investigative toxicology in the pharmaceutical industry. Nat Rev Drug Discov. (2023) 22:317–35. doi: 10.1038/s41573-022-00601-z 36781957 PMC9924869

[64] Vamathevan J Clark D Czodrowski P Dunham I Ferran E Lee G . Applications of machine learning in drug discovery and development. Nat Rev Drug Discov. (2019) 18:463–77. doi: 10.1038/s41573-019-0024-5 30976107 PMC6552674

[65] Lee H Kim J Kim J Lee Y . Recent advances in AI-based toxicity prediction for drug discovery. Front Chem. (2025) 13:1632046. doi: 10.3389/fchem.2025.1632046 40698059 PMC12279745

[66] El-Sayes N Vito A Mossman K . Tumor heterogeneity: A great barrier in the age of cancer immunotherapy. Cancers. (2021) 13:806. doi: 10.3390/cancers13040806 33671881 PMC7918981

[67] Minh D Wang HX Li YF Nguyen TN . Explainable artificial intelligence: A comprehensive review. Artif Intell Rev. (2022) 55:3503–68. doi: 10.1007/s10462-021-10088-y30311153

[68] Gilpin LH Bau D Yuan BZ Bajwa A Specter M Kagal L . (2018). “ Explaining explanations: An overview of interpretability of machine learning”, in: 2018 IEEE 5th International Conference on Data Science and Advanced Analytics (DSAA) (Piscataway, New Jersey), 80–9. doi: 10.1109/DSAA.2018.00018

[69] Algorri M Acharya A Bernstein J Cauchon NS Chen XH Huynh-Ba K . Meeting report: Advancing accelerated regulatory review with Real-Time Oncology Review (RTOR), Project Orbis, and the Product Quality Assessment Aid. AAPS Open. (2022) 8:19. doi: 10.1186/s41120-022-00066-1 36530577 PMC9734574

[70] Olawade DB Clement David-Olawade A Adereni T Egbon E Teke J Boussios S . Integrating AI into cancer immunotherapy—A narrative review of current applications and future directions. Diseases. (2025) 13:24. doi: 10.3390/diseases13010024 39851488 PMC11764268

[71] Boehm KM Khosravi P Vanguri R Gao J Shah SP . Harnessing multimodal data integration to advance precision oncology. Nat Rev Cancer. (2022) 22:114–26. doi: 10.1038/s41568-021-00408-3 34663944 PMC8810682

[72] Yoo W . Precision oncology in the age of AI: Lessons from AI-driven drug discovery and clinical translation. BJC Rep. (2026) 4:18. doi: 10.1038/s44276-026-00221-1 41986665 PMC13083990

[73] Qiu S Chen J Wu T Li L Wang G Wu H . CAR-Toner: An AI-driven approach for CAR tonic signaling prediction and optimization. Cell Res. (2024) 34:386–8. doi: 10.1038/s41422-024-00936-1 38351128 PMC11061301

[74] Nicora G Vitali F Dagliati A Geifman N Bellazzi R . Integrated multi-omics analyses in oncology: A review of machine learning methods and tools. Front Oncol. (2020) 10:1030. doi: 10.3389/fonc.2020.01030 32695678 PMC7338582

[75] Acharya D Mukhopadhyay A . A comprehensive review of machine learning techniques for multi-omics data integration: Challenges and applications in precision oncology. Briefings Funct Genomics. (2024) 23:549–60. doi: 10.1093/bfgp/elae013 38600757

[76] Zheng Y Liu Y Yang J Dong L Zhang R Tian S . Multi-omics data integration using ratio-based quantitative profiling with Quartet reference materials. Nat Biotechnol. (2024) 42:1133–49. doi: 10.1038/s41587-023-01934-1 37679543 PMC11252085

[77] Wen Y Ma L Ju C . Recent insights into the pathogenesis and therapeutic targets of chronic liver diseases. eGastroenterology. (2023) 1:e10100. doi: 10.1136/egastro-2023-100020 38074919 PMC10704956

[78] Khan SA Martínez-de-Morentin X Alsabbagh AR Maillo A Lagani V Gomez-Cabrero D . Multimodal foundation transformer models for multiscale genomics. Nat Methods. (2025). doi: 10.1038/s41592-025-02918-6 41326819

[79] Huang Q Wu S Ou Z Gao Y . Computational pathology: A comprehensive review of recent developments in digital and intelligent pathology. Intelligent Oncology, (2025) 1(2):139–59. doi: 10.1016/j.intonc.2025.03.004

[80] Waqas A Tripathi A Ramachandran RP Stewart PA Rasool G . Multimodal data integration for oncology in the era of deep neural networks: A review. Frontiers in Artificial Intelligence, (2024) 7:1408843. doi: 10.3389/frai.2024.1408843 PMC11308435 39118787

[81] Van Veldhuizen V Botha V Lu C Cesur ME Lipman KG de Jong ED . Foundation models in medical imaging: A review and. outlook arXiv preprint arXiv:2506.09095. (2025). doi: 10.48550/arXiv.2506.09095

[82] Azad B Azad R Eskandari S Bozorgpour A Kazerouni A Rekik I . Foundational models in medical imaging: A comprehensive survey and future vision. arXiv preprint arXiv:2310.18689. (2023). doi: 10.48550/arXiv.2310.18689

[83] Andrade-Miranda G Jaouen V Tankyevych O Cheze Le Rest C Visvikis D Conze PH . Multi-modal medical Transformers: A meta-analysis for medical image segmentation in oncology. Comput Med Imaging Graphics. (2023) 110:102308. doi: 10.1016/j.compmedimag.2023.102308 37918328

[84] Palazzo G Mangili P Deantoni C Fodor A Broggi S Castriconi R . Real-world validation of Artificial Intelligence-based Computed Tomography auto-contouring for prostate cancer radiotherapy planning. Phys Imaging Radiat Oncol. (2023) 28:100501. doi: 10.1016/j.phro.2023.100501 37920450 PMC10618761

[85] Olawade DB Oisakede EO Bello OJ Analikwu CC Egbon E Ojo A . Digital twins in oncology: From predictive modelling to personalised treatment strategies. Critical Reviews in Oncology/Hematology, (2026) 220:105171. doi: 10.1016/j.critrevonc.2026.105171 41621635

[86] Hosny A Bitterman DS Guthier CV Qian JM Roberts H Perni S . Clinical validation of deep learning algorithms for radiotherapy targeting of non-small-cell lung cancer: an observational study. Lancet Digital Health. (2022) 4:e657–66. doi: 10.1016/S2589-7500(22)00129-7 36028289 PMC9435511

[87] Zhu S Gilbert M Chetty IJ Siddiqui F . The 2021 landscape of FDA-approved artificial intelligence/machine learning-enabled medical devices: An analysis of the characteristics and intended use. International Journal of Medical Informatics, (2022) 165:104828. doi: 10.1016/j.ijmedinf.2022.104828 35780651

[88] Kuchana SK Repalle UK Alahari NV Kondamuri M Manduva SK Vanguru RV . Artificial Intelligence in Oncology: A comprehensive cross-cancer translational readiness analysis across 18 Malignancies. Cancers (Basel). (2026) 18:1543. doi: 10.3390/cancers18101543 42192906 PMC13204750

[89] Kneepkens E Bakx N van der Sangen M Theuws J van der Toorn PP Rijkaart D . Clinical evaluation of two AI models for automated breast cancer plan generation. Radiat Oncol. (2022) 17:25. doi: 10.1186/s13014-022-01993-9 35123517 PMC8817521

[90] Ankolekar A Boie S Abdollahyan M Gadaleta E Hasheminasab SA Yang G . Advancing breast, lung, and prostate cancer research with federated learning: A systematic review. NPJ Digital Med. (2025) 8:314. doi: 10.1038/s41746-025-01591-5 40425787 PMC12117161

[91] Mir BA Abbas SR Lee SW . Federated learning in healthcare ethics: A systematic review of privacy-preserving and equitable medical AI. Healthcare, (2026) 14(3):306. doi: 10.3390/healthcare14030306 PMC12896918 41682156

[92] Zerka F Barakat S Walsh S Bogowicz M Leijenaar RTH Jochems A . Systematic review of privacy-preserving distributed machine learning from federated databases in health care. JCO Clin Cancer Inform. 4:184–200. doi: 10.1200/CCI.19.00047openaccess.city.ac PMC7113079 32134684

[93] Gill JK Chetty M Lim S Hallinan J . Large language model based framework for automated extraction of genetic interactions from unstructured data. PLoS One. (2024) 19:e0303231. doi: 10.1371/journal.pone.0303231 38771886 PMC11108146

[94] Xu M Chen Z Zheng J Zhao Q Yuan Z . Artificial intelligence-aided optical imaging for cancer detection and diagnosis. Semin Cancer Biol. (2023) 94:62–80. doi: 10.1016/j.semcancer.2023.06.003 37302519

[95] Krewski D Andersen ME Mantus E Zeise L . Toxicity testing in the 21st century: A vision and a strategy. J Toxicol Environ Health Part B. (2010) 13:51–138. doi: 10.1080/10937404.2010.483176 20574894 PMC4410863

[96] Mak K-K Pichika MR . Artificial intelligence in drug development: Present status and future prospects. Drug Discov Today. (2019) 24:773–80. doi: 10.1016/j.drudis.2018.11.014 30472429

[97] Gaulton A Hersey A Nowotka M Bento AP Chambers J Mendez D . The chEMBL database in 2017. Nucleic Acids Res. (2017) 45:D945–54. doi: 10.1093/nar/gkw1074 27899562 PMC5210557

[98] Myung Y de Sá AGC Ascher DB . Deep-PK: Deep learning for small-molecule pharmacokinetic and toxicity prediction. Nucleic Acids Res. (2024) 52:W469–75. doi: 10.1093/nar/gkae299 38634808 PMC11223837

[99] Zhang Y Wang X Li C colleagues . Helix-ADMET: Self-supervised multitask learning for ADMET prediction. Briefings Bioinf. (2022) 23:bbac455. doi: 10.1093/bib/bbac455 36274234

[100] Zhang L Ai H Chen W Yin Z Hu H Zhu J . CarcinoPred-EL and admetSAR 2.0: New tools for toxicity and ADMET prediction. Nucleic Acids Res. (2019) 47:W5–W11. doi: 10.1093/nar/gkz318 31053845 PMC6648356

[101] Yang F Zhang Q Ji X Zhang Y Li W Peng S . Machine learning applications in drug repurposing. Interdiscip Sci. (2022) 14(1):15–21. doi: 10.1007/s12539-021-00487-8 PMC8783773 35066811

[102] Thompson RF Valdes G Fuller CD Carpenter CM Morin O Aneja S . Artificial intelligence in radiation oncology: A specialty-wide disruptive transformation? Radiotherapy Oncol. (2018) 129:421–6. doi: 10.1016/j.radonc.2018.11.007 29907338 PMC9620952

[103] Xu Y Su G-H Ma D Xiao Y Shao Z-M Jiang Y-Z . Technological advances in cancer immunity: From immunogenomics to single-cell analysis and artificial intelligence. Signal Transduction Targeted Ther. (2021) 6:312. doi: 10.1038/s41392-021-00729-7 34417437 PMC8377461

[104] Prelaj A Miskovic V Zanitti M Tròvo F Genova C Viscardi G . Artificial intelligence for predictive biomarker discovery in immuno-oncology: A systematic review. Ann Oncol. (2024) 35:29–65. doi: 10.1016/j.annonc.2023.10.001 37879443

[105] Xie J Luo X Deng X Tang Y Tian W Cheng H . Advances in artificial intelligence to predict cancer immunotherapy efficacy. Front Immunol. (2023) 13:1076883. doi: 10.3389/fimmu.2022.1076883 36685496 PMC9845588

[106] Rassy E Assi T Rizkallah J Kattan J Pavlidis N . The role of immunotherapy in cancer treatment: Challenges and opportunities. Crit Rev Oncology/Hematology. (2021) 157:103166. doi: 10.1016/j.critrevonc.2020.103166 33254039

[107] Gómez-Bombarelli R Wei JN Duvenaud D Hernández-Lobato JM Sánchez-Lengeling B Sheberla D . Automatic chemical design using a data-driven continuous representation of molecules. ACS Cent Sci. (2018) 4(2):268–76. doi: 10.1021/acscentsci.7b00572 PMC5833007 29532027

[108] Basu T Engel-Wolf S Menzer O . The ethics of machine learning in medical sciences: Where do we stand today? Indian J Dermatol. (2020) 65:358–64. doi: 10.4103/ijd.IJD_419_20 PMC7640783 33165392

[109] Sandfort V Yan K Pickhardt PJ Summers RM . Data augmentation using generative adversarial networks (CycleGAN) to improve generalizability in medical imaging. Sci Rep. (2019) 9:16884. doi: 10.1038/s41598-019-52737-x 31729403 PMC6858365

[110] Fang C Zhou P Zhang X He Y Yang Q . Artificial intelligence in oncology drug development and management: A precision medicine perspective. Front Oncol. (2025) 15:1609827. doi: 10.3389/fonc.2025.1609827 41426328 PMC12711520

[111] Tsuji S Hase T Yachie-Kinoshita A Nishino T Ghosh S Kikuchi M . Artificial intelligence-based computational framework for drug-target prioritization and inference of novel repositionable drugs for Alzheimer’s disease. Alzheimer's Res Ther. (2021) 13:92. doi: 10.1186/s13195-021-00826-3 33941241 PMC8091739

[112] Grebner C Matter H Kofink D Wenzel J Schmidt F Hessler G . Application of deep neural network models in drug discovery programs. ChemMedChem. (2021) 16:3772–86. doi: 10.1002/cmdc.202100418 34596968

[113] Schraagen JM van Diggelen J . A brief history of the relationship between expertise and artificial intelligence. In: Germain ML Grenier RS . (Eds.), Expertise at work. Palgrave Macmillan. (2021). doi: 10.1007/978-3-030-64371-3_8

[114] Jarrahi MH . Artificial intelligence and the future of work: Human–AI symbiosis in organizational decision making. Business Horizons. (2018) 61:577–86. doi: 10.1016/j.bushor.2018.03.00742574925

[115] Mak K-K Pichika MR . Artificial intelligence in drug discovery and development. Drug Discov Today. (2019) 24:773–80. doi: 10.1016/j.drudis.2018.11.014 30472429

